# Single-nucleus ribonucleic acid-sequencing and spatial transcriptomics reveal the cardioprotection of Shexiang Baoxin Pill (SBP) in mice with myocardial ischemia-reperfusion injury

**DOI:** 10.3389/fphar.2023.1173649

**Published:** 2023-05-09

**Authors:** Wenyong Lin, Xin Chen, Dongyuan Wang, Ruixia Lu, Chunling Zhang, Zhenchao Niu, Jie Chen, Xiaofen Ruan, Xiaolong Wang

**Affiliations:** ^1^ Branch of National Clinical Research Center for Chinese Medicine Cardiology, Shuguang Hospital Affiliated to Shanghai University of Traditional Chinese Medicine, Shanghai, China; ^2^ Cardiovascular Research Institute of Traditional Chinese Medicine, Shuguang Hospital Affiliated to Shanghai University of Traditional Chinese Medicine, Shanghai, China; ^3^ Shanghai Innovation Center of TCM Health Service, Shanghai University of Traditional Chinese Medicine, Shanghai, China

**Keywords:** Shexiang Baoxin Pill, single-nucleus RNA sequencing, spatial transcriptomics, traditional Chinese medicine, myocardial ischemia reperfusion injury

## Abstract

**Aim:** The Shexiang Baoxin Pill (SBP) has been extensively used to treat cardiovascular diseases in China for four decades, and its clinical efficacy has been widely approved. However, the mechanism by which this is achieved remains largely unexplored. Research attempting to understand the underlying mechanism is ongoing, but the findings are controversial. Here, we aimed to explore the possible mechanism of SBP in myocardial ischemia-reperfusion (I/R) injury using heart single-nucleus and spatial ribonucleic acid (RNA) sequencing.

**Methods:** We established a murine myocardial I/R injury model in C57BL/6 mice by ligating and recanalizing the left coronary artery anterior descending branch. Subsequently, single-nucleus RNA-seq and spatial transcriptomics were performed on mice cardiac tissue. We initially assessed the status of cell types and subsets in the model administered with or without SBP.

**Results:** We used single-nucleus RNA sequencing to comprehensively analyze cell types in the cardiac tissue of sham, I/R, and SBP mice. Nine samples from nine individuals were analyzed, and 75,546 cells were obtained. We classified the cells into 28 clusters based on their expression characteristics and annotated them into seven cell types: cardiomyocytes, endothelial cells, fibroblasts, myeloid cells, smooth muscle cells, B cells, and T cells. The SBP group had distinct cellular compositions and features than the I/R group. Furthermore, SBP-induced cardioprotection against I/R was associated with enhanced cardiac contractility, reduced endocardial cell injury, increased endocardial-mediated angiogenesis, and inhibited fibroblast proliferation. In addition, macrophages had active properties.

**Conclusion:** SBP improves the early LVEF of I/R mice and has a cardioprotective effect. Through sequencing analysis, we observed that SBP can increase the gene expression of *Nppb* and *Npr3* in the infarct area of the heart. *Npr3* is related to vascular generation mediated by endocardial cells and requires further research. In addition, SBP increases the number of fibroblasts, inhibits the expression of genes related to fibroblast activation and proliferation, and increases the transformation of endothelial cells into fibroblasts. These findings will help to indicate directions for further research.

## Graphical Abstract

**Figure:**
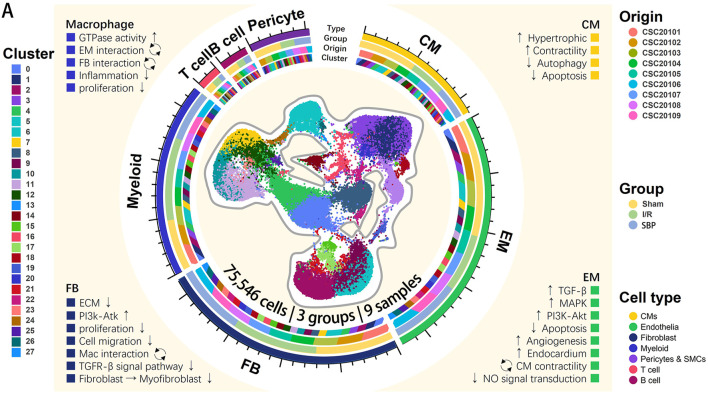
Single-cell landscape of SBP treatments in a mouse model of myocardial I/R injury. A total of 75,546 cells from nine mouse cardiac tissues in 3 different groups are projected by UMAP plot. The four colored tracks (from inside to outside) indicate cluster, origin ID, group and cell type. The axis outside the circular plot depicts the scale of the total cell number for each class. The text in each of the four corners show an overview of the role of SBP on cardiomyocytes, endothelial cells, fibroblasts, and macrophages respectively. Legends denote the origin ID, group, cell type and cluster.

## 1 Introduction

Myocardial infarction (MI) is a major cause of morbidity and mortality associated with cardiovascular diseases (Benjamin et al., 2018). Percutaneous coronary intervention is the most effective method to ameliorate myocardial injury (Cohen et al., 2010). Sudden recanalization is inherently complicated by increasing irreversible injury to the myocardium despite blood flow restoration, causing cardiac and microvascular dysfunction (Fröhlich et al., 2013). The links between myocardial I/R injury and coronary microvascular diseases have attracted increasing attention. However, clinical drugs have limited efficacy in treating I/R. Therefore, identifying new drugs to treat myocardial I/R injury is important.

Shexiang Baoxin Pill (SBP) is a traditional Chinese medicine (TCM) compound made by the ancient prescription Suhexiang pill through modern pharmacology technology, which can activate Yang and benefit vital energy in resuscitation with aromatics according to Yin and Yang theory in Chinese medicine (Ge et al., 2020; Zhao et al., 2021). SBP is approved by the Chinese Food and Drug Administration for use in the secondary prevention of coronary heart disease (CHD) and first aid in acute coronary syndrome (ACS) (Wen et al., 2018; Ge et al., 2020). After four decades of clinical application, SBP was first recorded in the Chinese pharmacopeia in 2015 and comprised seven materials: *Moschus* (She Xiang), *Calculus Bovis Artifactus* (Rengong Niuhuang), *Styrax* (Su Hexiang), *Ginseng Radix et Rhizoma* (Ren Shen), *Cinnamomi Cortex* (Rou Gui), *Bufonis Venenum* (Chan Su) and *Borneolum Syntheticum* (Bing Pian). SBP can preserve cardiac function by improving endothelial vascular function, inhibiting inflammation and antioxidative stress, restoring myocardial energy metabolism, and promoting angiogenesis (Fang et al., 2017; Huang et al., 2017; Lu et al., 2019; Zhang J et al., 2020; Guo et al., 2021). In the MUST study (Ge et al., 2020), SBP significantly reduced angina frequency and was associated with reduced major adverse cardiovascular events in patients with CHD. Due to the excellent therapeutic effect of SBP on CHD and ACS, its protective effect against myocardial I/R injury has attracted great attention. However, the underlying molecular mechanisms remain unclear.

Here, we aimed to preliminary describe ecological changes in various cells and potential targets in the heart after SBP intervention using single-nucleus RNA-sequencing (sNuc-seq) and spatial transcriptomics-sequencing (ST-seq). This study established a cellular landscape that provides evidence for clinical applications and support for further research.

## 2 Materials and methods

### 2.1 Materials

SBP was obtained from Shanghai Hutchison Pharmaceuticals Co., Ltd. (Shanghai, China; manufacturer’s batch number: 190307). The SBP quality control adhered to the specifications and test procedures described in the Pharmacopeia of the People’s Republic of China (Chinese Pharmacopeia Commission, 2015).

### 2.2 Animals

All animal experiments were approved and conducted in strict compliance with the Shanghai University of Traditional Chinese Medicine Institutional Animal Care and Use Committee guidelines (Permit Number: PZSHUTCM210913016). The Laboratory Animal Center of Shanghai University of Traditional Chinese Medicine supplied specific pathogen-free grade C57BL/6 mice (20–22 g, 8 weeks). The mice were housed in groups of five per cage with a controlled light/dark cycle of 12/12 h, temperature of 24°C ± 1°C, and humidity of 50%–70%. All animals were acclimatized for a week with water and food available *ad libitum*. Subsequently, mice were randomly assigned to three groups (n = 15): 1) sham (control), 2) I/R (model), and 3) SBP (drug intervention).

### 2.3 SBP preparation and treatment

The quality control of SBP is described in the Chinese Pharmacopeia. We applied the correction factor method (Nair and Jacob, 2016) to convert the adult dose of SBP to the equivalent dose in a mouse, and the correction factor (K_m_) was estimated by dividing the average body weight (kg) of the species by its body surface area (m^2^). For example, we assumed that the average adult body weight (bw) is 60 kg, the adult clinical dose of SBP is 135 mg/d (instruction manual recommended), the K_m_ for an adult is 37, and the K_m_ for a mouse is 3, we calculated the mouse equivalent dose as:

Mouse equivalent dose (mg/kg bw/d) = Adult dose (mg/kg bw/d) × Adult k_m_/Animal k_m_ = 135 × 60/37/3 = 27.75 mg/kg bw/d.

Therefore, the equivalent dose for a mouse was 27.75 mg/kg bw/d. This dose has exhibited cardioprotective efficacy without toxicity in previous studies (Yu et al., 2022). Furthermore, considering that the maximum daily volume of gavage in a mouse is approximately 0.01 mL/g bw, we set the SBP concentration to 2.775 mg/mL. SBP was dissolved in a vehicle containing a 0.5% aqueous sodium carboxymethyl cellulose solution. All animals were administered SBP solution or vehicle via oral gavage for at least seven consecutive days before use.

### 2.4 Modeling and grouping

The left anterior descending coronary artery (LAD) was ligated and recanalized to establish a myocardial I/R injury model. Briefly, mice were anesthetized with 4% isoflurane, and endotracheal intubation was performed and connected to a small animal ventilator with 1.5% isoflurane. An electrocardiogram was used to monitor the mice during the procedure in real-time to ensure that the experimental model was qualified. A left intercostal thoracotomy was performed on mice to induce I/R injury at the fourth intercostal space to expose the heart. For ligation, a 10-0 nylon suture was used to bind the LAD and PE-10 sterile tubes, which can block coronary blood flow and avoid irreversible damage to blood vessels and myocardium. The tube was removed after 30 min of ischemia. Observation (the ventricular anterior wall turns pallor) and electrocardiogram monitoring (ST-T segment elevation) were used to judge the success of coronary artery occlusion, and reversing these effects was a sign of successful reperfusion. The sham group underwent identical operation procedures without LAD ligation and recanalization. Postoperatively, penicillin sodium (80,000 U per mouse) was administered intraperitoneally to prevent postoperative infection. The reperfusion process was continued for 24 h. Both groups were sampled 24 h after reperfusion.

### 2.5 Quality assessment of SBP

Liquid chromatography conditions: The mobile phase was a gradient elution of 0.1% formic acid aqueous solution (C)—acetonitrile (D), with the following program: 0–1 min, 95% C-5% D; 1–6 min, 68% C-32% D; 6–12 min, 45% C-55% D; 12–19 min, 5% C-95% D; 19–22 min, 95% C-5% D. The flow rate was 0.3 mL/min, the column temperature was 45°C, and the injection volume was 3 μL.

Mass spectrometry conditions: The ion source was HESI; positive ion spray voltage: 3.5 kV, negative ion spray voltage: 2.5 kV; auxiliary gas flow rate 12 L/min, sheath gas flow rate 40 L/min, capillary temperature 330°C, auxiliary gas heating temperature 300°C, collision energy (CE) 50 eV. The detection mode was Full MS, with a Full MS resolution of 70,000 and a scan range of m/z 80–1,200: Bufalin, Chenodeoxycholic acid, Cholic acid, Cinnamic acid, Cinnamic aldehyde, Cinobufagin, Deoxycholic acid, Gamabufalin, Ginsenoside Rb1, Ginsenoside Rg1, Ginsenoside Rb2, Ginsenoside Rb3, Ginsenoside Rd, Ginsenoside Re, Ginsenoside Rf, Hyodeoxycholic acid, Ursodeoxycholic acid. (Results are provided by Shanghai Hutchison Pharmaceuticals Co., Ltd., China, Supplementary Table S1, Supplementary Figures S1–11).

### 2.6 Cardiac function measurement

An ultrasound cardiogram (UCG) was performed to evaluate cardiac function in mice. Briefly, mice were anesthetized with 2% isoflurane during UCG detection. A short-axis view of the left ventricle was obtained to measure five continuous cardiac cycles. M-mode tracing was performed to record the left ventricular end-diastolic dimension (LVEDD), left ventricular end-systolic dimension (LVESD), left ventricular diastolic posterior wall thickness, left ventricular end-diastolic volume (LVEDV), and left ventricular end-diastolic volume (LVESV), which were calculated automatically using a computer based on LVEDD and LVESD. Left ventricular ejection fraction (LVEF) = 100% × [(LVEDV–LVESV)/LVEDV].

### 2.7 Enzyme-linked immunosorbent assay

Whole blood was clotted at room temperature for 2 h and centrifuged at 1,500 *g* for 10 min. The lactate dehydrogenase (LDH) and the cardiac troponin I (cTnI) level was measured by enzyme-linked immunosorbent assay (ELISA) kits (Nanjing Jiancheng, Jiangsu, China). According to the manufacturer’s instructions.

### 2.8 Hematoxylin-eosin and Masson’s trichrome staining

The cardiac tissues were fixed in buffered paraformaldehyde and embedded in paraffin for Hematoxylin-Eosin (H&E, Solarbio, Beijing, China) and Masson’s trichrome (Solarbio, Beijing, China) staining. Histological changes in the cardiac tissues of different groups were investigated using H&E and Masson’s trichrome staining of 5 μm thick tissue sections via optical microscopy at 200 and 1,000 μm magnification.

### 2.9 Infarct size measurement

2,3,5-triphenyltetrazolium chloride (TTC, Sigma-Aldrich, United States) staining was used to demarcate non-ischemic area (NIA, red) and area at risk (AAR, white). Briefly, the cardiac tissues were frozen for 20 min at −80°C, then sectioned the tissues into 0.5 mm slices and stained in 2% TTC solution for 15 min avoid to light. The sections were immersed in 4% paraformaldehyde overnight. Image J (National Institutes of Health, United States) was used to calculate the infarct size.

### 2.10 Frozen tissue nuclear dissociation for sNuc-seq

Mice hearts were harvested and frozen after sacrificing them. Frozen tissue samples were cut into pieces <0.5 cm and homogenized using a glass Dounce tissue grinder (Sigma, cat. no. D8938). The tissue was homogenized 25 times with pestle A and 25 times with pestle B in 2 mL of ice-cold nuclei EZ lysis buffer. The sample was then incubated in ice for 5 min with 3 mL of cold EZ lysis buffer. Nuclei were centrifuged at 500 *g* for 5 min at 4°C, washed with 5 mL ice-cold EZ lysis buffer, and incubated on ice for 5 min. After centrifugation, the nuclear pellet was washed with 5 mL nuclei suspension buffer [NSB; consisting of 1% PBS, 0.01% BSA, and 0.1% RNase inhibitor (Clontech, cat. no. 2313A)]. Isolated nuclei were resuspended in 2 mL NSB, filtered through a 35 μm cell strainer (Corning-Falcon, cat. no. 352235) and counted. A final concentration of 1,000 nuclei per μl was used for loading onto a 10x channel.

### 2.11 10x library preparation and sNuc-seq

We used a previously described standard procedure (Yoon et al., 2019). Briefly, beads with a unique molecular identifier (UMI) and cell barcodes were loaded close to saturation such that each cell was paired with a bead in a gel beads-in-emulsion. After exposure to the cell lysis buffer, polyadenylated RNA molecules were hybridized into the beads. Beads were retrieved in a single tube for reverse transcription. For complementary deoxyribonucleic acid (cDNA) synthesis, each cDNA molecule was tagged on the 5′ end (that is, the 3′ end of a messenger RNA transcript) with UMI and a cell label indicating its cell of origin. Briefly, 10× beads were subjected to second-strand cDNA synthesis, adaptor ligation, and universal amplification. Sequencing libraries were prepared using randomly interrupted whole-transcriptome amplification products to enrich the 3′ end of the transcripts linked to the cell barcode and UMI. All remaining procedures, including library construction, were performed according to the standard manufacturer’s protocol (Chromium Single Cell 3ʹ v3). Sequencing libraries were quantified using a High Sensitivity DNA Chip (Agilent) on a Bioanalyzer 2100 and Qubit high-sensitivity DNA Assay (Thermo Fisher Scientific). The libraries were sequenced on NovaSeq6000 (Illumina) using 2 × 150 chemistry.

### 2.12 sNuc-seq data processing

We applied fastp with default parameter filtering the adaptor sequence and removed the low quality reads to achieve the clean data. Then the feature-barcode matrices were obtained by aligning reads to the mouse genome (mm10) using CellRanger v6.1.1.

We applied the down sample analysis among samples sequenced according to the mapped barcoded reads per cell of each sample and finally achieved the aggregated matrix. Cells contained over 200 expressed genes and mitochondria UMI rate below 10% passed the cell quality filtering and mitochondria genes were removed in the expression table. Seurat package (version: 4.0.3, https://satijalab.org/seurat/) was used for cell normalization and regression based on the expression table according to the UMI counts of each sample and percent of mitochondria rate to obtain the scaled data. The fastMNN function (k = 5, d = 50, approximate = TRUE) in the R package scran (v1.12.1) was used to apply the mutual nearest-neighbour method to correct for batch effect among samples.

PCA was constructed based on the scaled data with top 2,000 high variable genes and top 10 principals were used for tSNE construction and UMAP construction. Utilizing graph-based cluster method, we acquired the unsupervised cell cluster result based the PCA top 10 principal and we calculated the marker genes by FindAllMarkers function with wilcox rank sum test algorithm under following criteria:1. lnFC > 0.25; 2. *p*-value < 0.05; 3. min. pct > 0.1. In order to identify the cell type detailed, the clusters of same cell type were selected for re-tSNE analysis, graph-based clustering and marker analysis.

### 2.13 Tissue enrichment quantification for clusters

We used the R_o/e_ ratio to determine the preference of each cluster in the sham, I/R, and SBP groups for different tissues to quantify the degree of tissue enrichment, as previously described (Zhang et al., 2018; Zhang L et al., 2020). R_o/e_ is the ratio of the number of observed cells to the number of cells expected for a particular cell population and tissue. Identifying R_o/e_ can indicate whether cells of a particular cell population are enriched or depleted in a particular tissue, effectively quantifying the tissue preference for cell clusters.

### 2.14 Differential gene expression (DEG) analysis

We used DESeq2 (v. 1.20) to explore the statistical power of the replicates to identify DEGs among the three groups (Love et al., 2014). DEGs with |log2FC| > 0.25 and Q value ≤ 0.05 were significantly different expressed genes. DEGs were analyzed separately for each cluster and subcell type. Hence, we can evaluate the fold change and adjust *p*-value of DEGs between SBP and I/R samples to identify SBP target genes and cell populations.

### 2.15 Function enrichment analysis

Gene ontology (GO) analysis was performed to elucidate the biological implications of unique genes in the significant or representative profiles of DEGs. GO annotations were downloaded from NCBI (http://www.ncbi.nlm.nih.gov/), UniProt (http://www.uniprot.org/), and GO (http://www.geneontology.org/). Fisher’s exact test was applied to identify significant GO categories, and FDR was used to correct *p*-values. According to the Kyoto Encyclopedia of Genes and Genomes database, pathway analysis was used to determine the significant pathways of the differential genes. Fisher’s exact test was used to select the significant pathway, and the *p*-value and FDR defined the significance threshold.

### 2.16 Tissue processing and Visium data genera for ST RNA-seq

The tissue was gently washed with cold PBS, cut into 4–6 mm^3^ pieces, and the cut side was placed into a plastic mold. The OCT-filled mold was snap-frozen in chilled isopentane. The sample was sent for execution, and cryosections were cut at a 10-µm thickness (Leica, cm 1950). Sections were placed on chilled Visium tissue optimization slides (catalog no. 3000394, 10x Genomics) and Visium spatial gene expression slides (catalog no. 2000233, 10x Genomics) and stuck firmly by warming the back of the slide. Tissue sections were fixed in chilled methanol and stained according to the Visium spatial gene expression user guide (catalog no. CG000239 Rev A, 10x Genomics) or Visium spatial tissue optimization user guide (catalog no. CG000238 Rev A, 10x Genomics). The tissue was permeabilized for 20 min for gene expression samples, which was selected as the optimal time based on tissue optimization time-course experiments. Brightfield histological images were obtained using a ×20 objective on a Pannoramic MIDI scanner (3D HISTECH). Raw images were stitched together using pannoramic MIDI and exported as. tiff files using low- and high-resolution settings. Fluorescent images were captured with a TRITC filter using a ×20 objective for tissue optimization.

### 2.17 10x library preparation and ST-seq

Libraries were prepared according to the Visium spatial gene expression user guide (CG000239: https://assets.ctfassets.net/an68im79xiti/3pyXucRaiKWcscXy3cmRHL/a1ba41c 77cbf60366202805ead8f64d7/CG0 00239_VisiumSpatialGeneExpression_UserGuide_Rev_A.pdf). They were loaded at 300 p.m. and sequenced on a NovaSeq 6000 System (Illumina) using a NovaSeq S4 Reagent Kit (200 cycles, catalog no. 20027466, Illumina) at a sequencing depth of approximately 250 400 10 106 read pairs per sample. Sequencing was performed using the following read protocol: read 1, 28 cycles; i7 index read, 10 cycles; i5 index read, 10 cycles; and read 2, 91 cycles.

### 2.18 ST-seq data processing

Raw FASTQ files and histology images were processed using the Space Ranger software v.1.2.2, which uses STAR (Dobin et al., 2013) for genome alignment against the Cell Ranger hg38 reference genome refdata-cellranger-GRCh38-3.0.0, available at http://cf.10xgenomics.com/supp/cell-exp/refdata-cellranger-GRCh38-3.0.0.tar.gz. The per-spot quality metrics were evaluated using the SeuratV4.0 R Bioconductor package. We excluded UMI with fewer than 200 detected spots.

### 2.19 Identification of cell types and subtypes via dimensional reduction and cluster analysis

Gene expression from each voxel was normalized using sctransform (Hafemeister and Satija, 2019) in Seurat, which uses regularized negative binomial models to account for technical artifacts while preserving biological variance. The top 30 principal components were then calculated and used to construct the KNN graph. The Louvain (Blondel et al., 2008) algorithm was used to cluster voxels. The clusters were visualized on a 2-dimensional map produced using UMAP (McInnes et al., 2018). We used the Wilcoxon rank-sum test for each cluster to identify significant DEGs compared with the remaining clusters. SingleR was used to identify cell types.

### 2.20 Statistical analysis

All values are expressed as mean ± standard error of mean (SEM). Unpaired two-tailed student's t tests were used for comparisons between two values. For multiple comparisons between groups of three, one-way ANOVA was used if variances were equal; otherwise, Dunnett’s T3 test was utilized. Values of *p* less than 0.05 were considered statistically significant.

## 3 Results

### 3.1 SBP has a cardioprotective effect on mice with myocardial I/R injury

**FIGURE 1:**
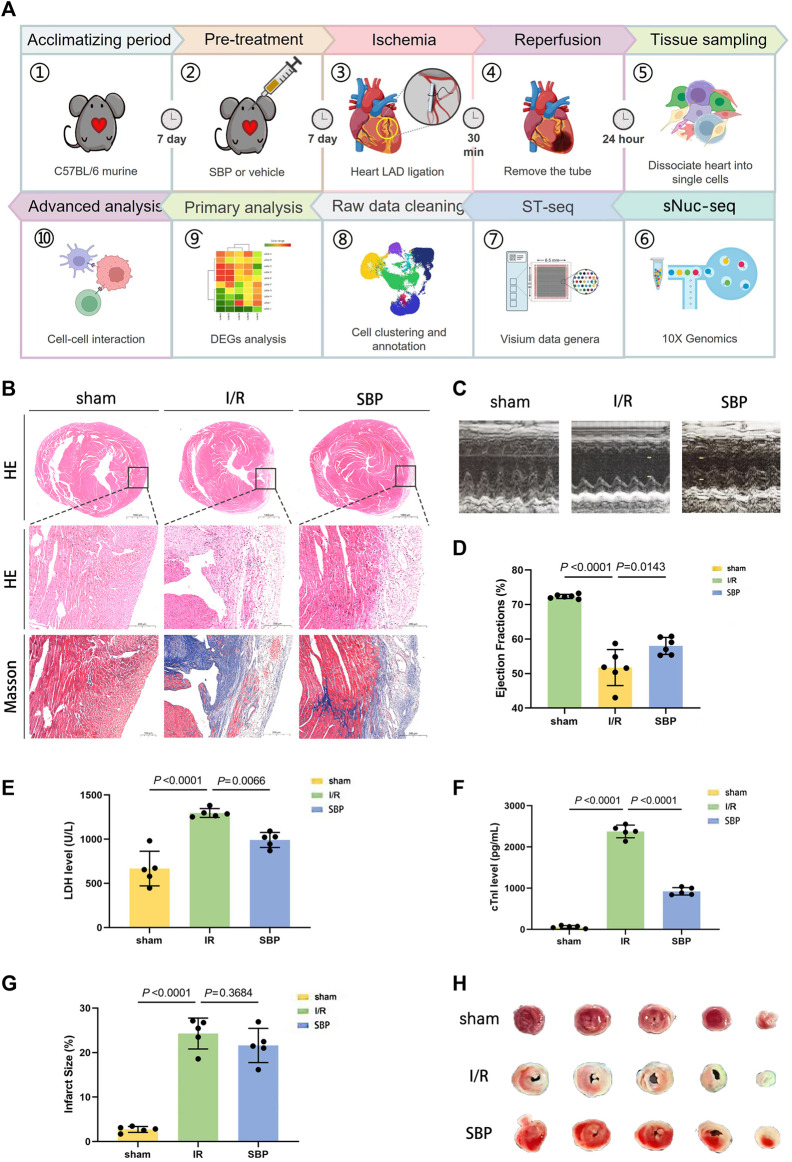
Experiment protocol and SBP cardioprotective effect. **(A)** Schematic representation of the experimental design. C57BL/6 mice were ligated and recanalized with LAD after intragastric administration for 1 week, and cardiac single cells were taken 24 h later for sNuc-seq and ST-seq. **(B)** H&E and Masson’s staining for cardiac tissue pathological sections in each group (Top: H&E staining, the bar equals 1,000 μm, Mid: H&E staining, the bar equals 200 μm, Bottom: Masson’s staining, the bar equals 200 μm). **(C)** M-mode ultrasound detection of left ventricular ejection fraction. **(D)** Quantitative assessment of left ventricular ejection fraction (n = 5, sham: 72.35% ± 0.60%; I/R: 51.74% ± 5.22%; SBP: 58.02% ± 2.48%). **(E)** Mean serum level of LDH was detected by ELISA kit (n = 5, sham: 668.10 ± 196.7 U/L; I/R: 1297.00 ± 50.40 U/L; SBP: 991.4 ± 85.10 U/L). **(F)** Mean serum level of cTnI was detected by ELISA kit (n = 5, sham: 61.54 ± 32.30 pg/mL; I/R: 2377.00 ± 153.40 pg/mL; SBP: 923.90 ± 90.20 pg/mL). **(G)**: Representative images of TTC staining of heart sections. **(H)** Infarct size measurement of cardiac tissues (n = 5, sham: 2.73% ± 0.66%; I/R: 24.29% ± 3.48%; SBP: 21.60% ± 3.83%).

We first performed the standard experimental procedure on C57BL/6 mice to clarify whether SBP is equally effective in animal experiments as in humans (Figure 1A). Mice were sacrificed for H&E staining, Masson’s staining, and cardiac ultrasound after 24 h postoperatively. Histological analysis of the three groups revealed that the ventricular wall of mice in the I/R group was significantly thinner, with massive cell necrosis and inflammatory cell infiltration, compared to the same location in the sham group. Furthermore, the ventricular walls of mice in the SBP group were thicker, with less pronounced inflammatory infiltration and fibrosis than in the I/R group (Figure 1B). LVEF of the mice was measured using M-mode ultrasonography. After 24 h of reperfusion, LVEF was significantly decreased in the I/R group; however, it was significantly enhanced in the SBP group vs. I/R group (Figures 1C, D). Hence, these results suggest that SBP exerts a protective effect against myocardial I/R injury. Furthermore, there was a significant difference in enzyme activity between SBP-pretreated and I/R mice, as indicated by the difference in serum LDH and cTnI concentrations (Figures 1E, F). Figure 1F showed that the infarct size was significantly higher in the I/R group than in the sham group (I/R: 24.29% ± 3.48% vs. sham: 2.73% ± 0.66%). These results suggested that the I/R model was well established. Of note, pretreatment of SBP could not decrease the infarct size (SBP: 21.60% ± 3.83% vs. I/R: 24.29% ± 3.48%) (Figures 1G, H).

### 3.2 SNuc-seq reveals the landscape of heart in SBP pre-treated mice with I/R

**FIGURE 2:**
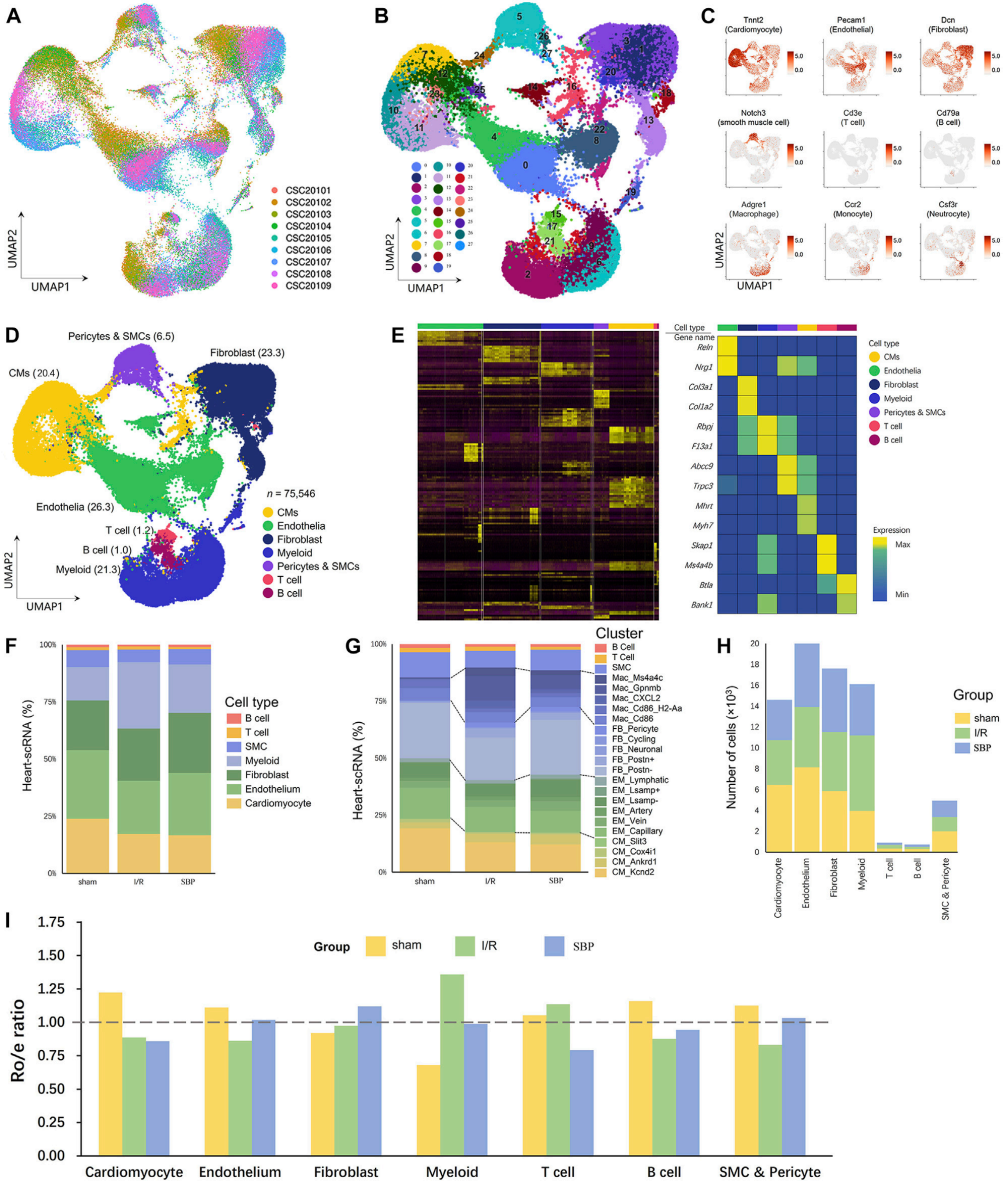
Myocardial I/R induces various changes in cardiac cells. **(A)** UMAP plot of cellular distribution from pooled sNuc-seq data of sham, I/R and SBP group cardiac tissues. (n = 3, CSC20101 - CSC20103: sham group, CSC20104 - CSC20106: I/R group, CSC20107–CSC20109: SBP group). **(B)** UMAP plot of cardiac cell populations (75,546 cells) with different treatments from three groups. Plot is color coded by 28 clusters (0–27). **(C)** UMAP Plot Shows traditional marker genes. **(D)** Eight cell types were annotated: myocardial cells (CM), endothelial cells (EM), fibroblasts (FB), myeloid cells, B cells, T cells, pericytes and smooth muscle cells (SMC) and unknown cells (other cells). **(E)** Heatmap showing log normalized UMIs for sNuc-seq analyses cells. Top and side bars indicate cell types. Highly variable genes for each type of cell are indicated (middle). **(F)** Proportion of cell types relative to total number of cells in each group. **(G)** Proportion of clusters relative to total number of cells in each group. **(H)** cell numbers in each group and each cell type. **(I)** Tissue preference of each cell type. The ratio of observed cell numbers to random expectation calculated by chi-square test (R_o/e_) was used to adjust cell-sampling biases for each sample. y-axis: average R_o/e_ across the 9 samples. R_o/e_> 1 (above the dashed line) indicates enrichment. R_o/e_ of the three groups are showed in different colors. Each bar plot represents one cell type.

We applied sNuc-seq from sham, I/R, and SBP mice mapping mouse cardiac cells in homeostasis and after I/R at the molecular resolution to better understand the effect of SBP on the composition of cell types and transcriptional patterns in cardiac tissues. We profiled nine samples from nine individuals, with all tissues collected from the part below the ligation. 10× Genomics was used to convert single-cell suspensions of sNuc-seq samples into barcoded libraries. We obtained 75,546 cells after quality control for subsequent analyses (Figure 2A). After quality control, normalization, batch effect correction, and clustering of sequencing data, we referred to several important studies for cell annotation (Hu et al., 2018; Kalucka et al., 2020; Litviňuková et al., 2020). In our study, we found 28 clusters of sequenced cells in cardiac tissues (Figure 2B) and identified all major cell types, including cardiomyocytes, fibroblasts, endothelial cells, macrophages, smooth muscle cells, T cells, and B cells: B cells (Marker: *Cd79a*, *Ms4a1*, and *Cd19*), cardiomyocyte (Marker: *Tnnc2*, *Ttn*, and *Pln*), endothelium (Marker: *Pecam1*, *Cdh5*, and *Npr3*), fibroblast (Marker: *Dcn*, *Fbln1*, and *Col1a2*). Myeloid cells, including macrophage (Marker: *C1qb*, *Adgre1*, and *Ms4a7*), monocyte (Marker: *Vcan*, *Ccr2*, and *Cd14*), neutrophil (Marker: *Csf3r*, *S100a8*, and *Retnlg*), smooth muscle cells (Marker: *Notch3*, *Pdgfrb*, and *Cspg4*) and T cells (Marker: *Cd3e*, *Cd2*, and *Cd3d*) (Figures 2C, D). Gene expression analysis showed distinct patterns of high expression of the 14 transcripts in each of these cell types (Figure 2E). Our findings are consistent with published studies on molecular profiles and cellular composition (Gladka et al., 2018; Farbehi et al., 2019; Zhang et al., 2019). We have attempted to integrate SBP data to establish a new cell landscape that might provide a new research strategy and evidence for the multi-target regulation of SBP.

### 3.3 SBP changes the cardiac cell composition

The cellular composition of the heart changes under pathological stress (Kehat and Molkentin, 2010), and disease generates new subpopulations of known cell types. We compared sNuc-seq datasets from sham, injured, and SBP-treated hearts to identify the novel molecular mechanism of SBP and depicted the cell abundance in each group and cluster (Figures 2F–H). We used the R_o/e_ ratio to determine the preference of each cell type for the tissue to quantify the extent of tissue enrichment (Figure 2I). Compared with the sham group, the I/R group showed a reduction in the number of myocardial cells, endothelial cells, smooth muscle cells, and B cells. SBP increased the number of endothelial cells and smooth muscle cells, while there was no significant change in the number of myocardial cells and B cells (vs. I/R group). We observed that the count and proportion of fibroblasts, myeloid cells, and T cells increased after the operation, but the effect of SBP was inconsistent for each of them. SBP further increased the proportion of fibroblasts but reduced the changes in myeloid and T cells. This reveals the cell populations targeted by SBP in early reperfusion stages and shows SBP’s potential to alter subpopulations of cell types and re-establish new cellular ecosystems in mouse cardiac tissue.

### 3.4 SBP enhances cardiomyocyte function

**FIGURE 3:**
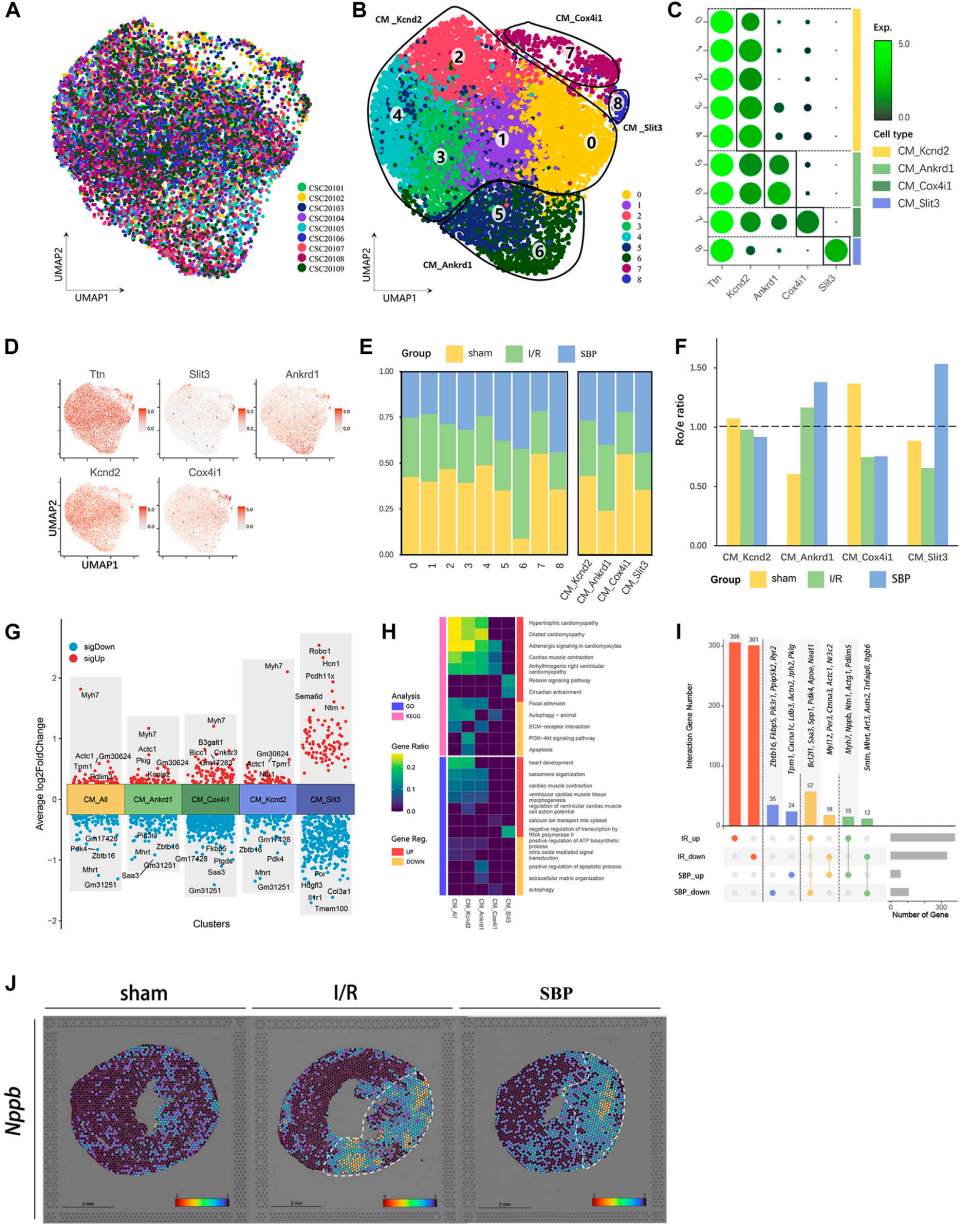
Single-cell characterization of cardiomyocyte in each group. **(A)** UMAP plot of cardiomyocyte distribution from pooled sNuc-seq data of each group tissues. **(B)** UMAP plot of cardiomyocyte populations with different treatments from three groups. Plot is color coded by nine clusters (0–8). **(C)** Dot plot showing fold-change (green to black) of marker genes associated with cell types and clusters. **(D)** UMAP Plot Shows marker genes. **(E)** Proportion and cell numbers of cell types and clusters relative to total number of cardiomyocytes in each group. **(F)** R_o/e_ shows tissue preference of each cell type in each group. **(G)** Differential gene analysis showing up and downregulated genes across four clusters and all cardiomyocytes (Y-axis: Average log2FoldChange, red: upregulated genes, blue: downregulated genes). **(H)** Heatmap plot shows the functional enrichment analysis of Gene Ontology (GO) and Kyoto Encyclopedia of Genes and Genomes (KEGG) on differential genes across each cluster and whole cells (SBP vs. I/R, Pink: KEGG, Blue: GO, Red: upregulated genes, Yellow: downregulated genes). **(I)** Upset plot describes different roles and target genes of SBP in the regulation of cardiomyocytes after myocardial I/R injury. The DEGs were partially listed in the plot (Red: Ineffective targets of SBP in I/R, Blue: I/R-independent targets, Yellow: Genes reversed by SBP, Green: Genes were further amplified by SBP). **(J)** Spatial transcriptome data predicted the spatial location of *Nppb* on heart sections.

We observed that SBP does not prevent cardiomyocyte loss. However, UCG and UMAP suggested cardiomyocyte heterogeneity in the SBP group. We extracted cardiomyocytes into nine new clusters (Figures 3A, B), followed by the marker gene, and merged these clusters into four cell subgroups, including CM_Kcnd2, CM_Ankrd1, CM_Cox4i1, and CM_Slit3 to further explore the source of these differences (Figures 3C, D). SBP showed a significant preference for CM_Ankrd1 and CM_ Slit3 (Figures 3E, F). Our differential analysis of total cardiomyocytes and cardiomyocyte subgroups revealed that SBP is mainly associated with hypertrophy and cardiac remodeling (*Myh7*, *Mhrt*, *Pdlim3*, *Actc1*, *Tpm1*, *Pkig*, *Zbtb16*); energy metabolism (*Pdk4*), inflammation (*Saa3*, *Fkbp5*), angiogenesis (*Ntn1*), regulation of aldosterone (*Cnksr3*), myocardial cell-mediated fibrosis (*Robo1*, *Col3a1*), regulation of ion channels (*Hcn1*, *Kcnip2*), and cell proliferation (*Sema6d*, *Hdgfl3*) (Figure 3G), CM_Slit3 might play roles in fibrosis and cardiac development by Slit3-Robo1 axis (Medioni et al., 2010; Gong et al., 2020), however, the function of CM_Slit3 needs further study. GO and Kyoto Encyclopedia of Genes and Genomes enrichment analyses were performed to identify the main biological functions and key pathways of the subgroups (Figure 3H). The upregulated genes were mainly enriched in cardiac hypertrophy, myocardial contraction, and myocardial contractile structures, while downregulated genes were mainly enriched in focal adhesion, autophagy, apoptosis, and the PI3K-AKT signaling pathway (SBP vs. I/R). In addition, we imported DEGs from the I/R group (I/R vs. sham) and the SBP group (SBP vs. I/R) into the upset plot to understand the relationship between SBP and DEGs, SBP regulated a total of 161 genes, of which 75 were reversed and 27 were further modulated. Additionally, 607 genes were unaffected by SBP, while 59 genes remained independent of the disease state (Figure 3I, Supplementary Table S3). We focused on 75 reversed genes and 27 genes amplified using SBP. Figure 3I shows that the expression of the *Nppb* gene is upregulated during the I/R process, and further upregulated by SBP. The *Nppb* gene encodes the BNP protein, so we mapped *Nppb* to its spatial location (Figure 3J). The expression of *Nppb* gene is mainly concentrated in the infarction area, BNP can inhibit cardiac remodeling and reduce cardiac load. These results suggested that SBP may play a cardioprotective role by promoting BNP secretion. In addition, SBP regulates 59 disease-independent genes, which warrants further studies. In conclusion, SBP could increase the LVEF (Figures 1C, D), DEGs and enrichment analysis highlighted strong upregulation of SBP with myocardial contraction. These data suggested that although SBP did not reduce the loss of cardiomyocytes, it might have a myocardial protective effect by enhancing the function of cardiomyocytes.

### 3.5 SBP promotes endocardial-mediated angiogenesis

**FIGURE 4:**
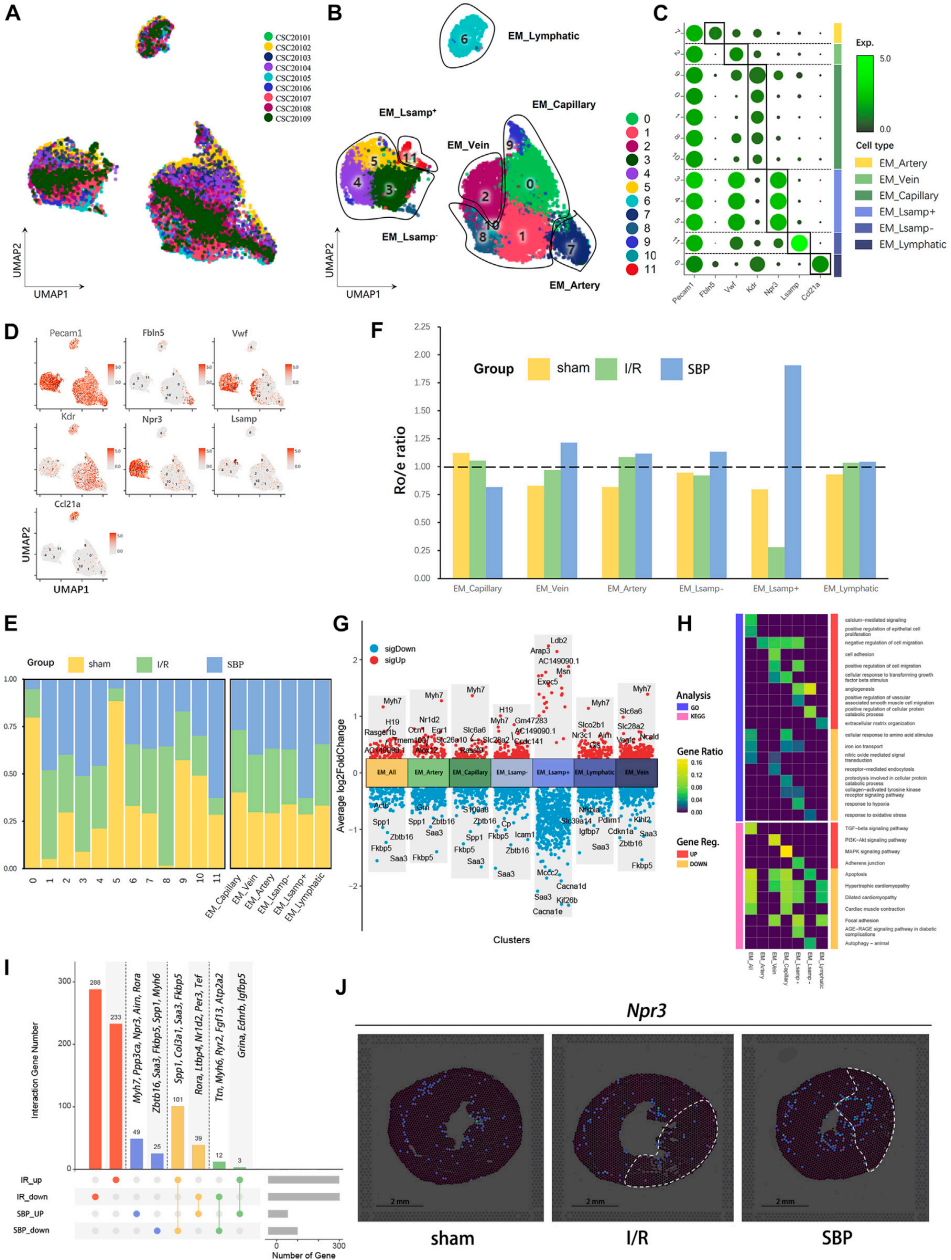
Single-cell characterization of endothelia cells in each group. **(A)** UMAP plot of endothelia cells distribution from pooled sNuc-seq data of each group tissues. **(B)** UMAP plot of endothelia cell populations with different treatments from three groups. Plot is color coded by 12 clusters (0–11). **(C)** Dot plot showing fold-change (green to black) of marker genes associated with cell types and clusters. **(D)** UMAP Plot Shows marker genes. **(E)** Proportion and cell numbers of cell types and clusters relative to total number of endothelia cells in each group. **(F)** R_o/e_ shows tissue preference of each cell type in each group. **(G)** Differential gene expression analysis showing up and downregulated genes across four clusters and all cardiomyocytes (Y-axis: Average log2FoldChange, red: upregulated genes, blue: downregulated genes). **(H)** Heatmap plot shows the functional enrichment analysis of GO and KEGG on differential genes across each cluster and whole cells (SBP vs. I/R). **(I)** Upset plot describes different roles and target genes of SBP in the regulation of endothelia cells after myocardial I/R injury. **(J)** Spatial transcriptome data predicted the spatial location of endocardium (*Npr3*) on heart sections.

Current mainstream studies suggest that the cardioprotective effect of SBP is mainly mediated by endothelial cells (Wang et al., 2021). In this study, 13,463 cells with endothelial characteristics were captured. We distinguished these cells into 12 clusters (Figures 4A, B) and annotated them using marker genes as capillary endothelial cells (EM_capillary), venous endothelial cells (EM_vein), arterial endothelial cells (EM_artery), lymphatic endothelial cells (EM_lymphatic), Lsmap^+^ endocardial cells (EM_ Lsamp^+^), and Lsmap^−^ endocardial cells (EM_Lsmap^−^) (Figures 4C, D). We discovered that the endothelial environment had changed 24 h after reperfusion, with SBP contributing to a decrease in capillaries and an increase in endocardial cells, whereas other cells did not change significantly (Figures 4E, F). Angiogenesis is a key part of the pathological process. Endothelial cells can be induced to proliferate and ensure blood and oxygen supply to the organ by stimulating molecules and signaling pathways in the pro-angiogenic process, thus accelerating injury repair (Sun et al., 2018). Our differential analysis of total endothelial cells and endothelium subgroups revealed that SBP is mainly associated with angiogenesis (*Ccn1*, *Arap3*, *Vegfc*, *Igfbp7*), cell proliferation and migration (*Egr1*, *Cdkn1a*, *Ldb2*, *Pdlim1*, *Kif26b*), lipid metabolism (*Egr1*, *Alox12*), cell death (*S100a8*, *Cp*), vascular permeability (*Msn*), inflammation (*Fkbp5*, *Saa3*, *Grn*, *Icam1*), statin transport (*Slco2b1*), and cardiac remodeling (*Spp1*, *Myh7*, *Slc6a6*, *Nr3c1*, *Airn*, *Gls*) (Figure 4G). Notably, SBP significantly upregulated endocardial-mediated angiogenesis (Figure 4H). In addition, we imported DEGs from the I/R group (I/R vs. sham) and the SBP group (SBP vs. I/R) into the upset plot to understand the relationship between SBP and DEGs, SBP regulated a total of 229 genes, of which 140 were reversed and 15 were further modulated. Additionally, 521 genes were unaffected by SBP, while 74 genes remained independent of the disease state (Figure 4I; Supplementary Table S4). In the spatial transcriptome sections, we discovered that *Npr3* expression gradually extended from the endocardium to the infarct area endocardial cells in the SBP group, but not in the I/R group (Figure 4J). These results suggest that SBP exerts a specific regulatory effect on the endocardium. Our study reports, for the first time, the effect of SBP on different endothelial cells, especially endocardial cells.

### 3.6 Effect of SBP on fibroblast during I/R

**FIGURE 5:**
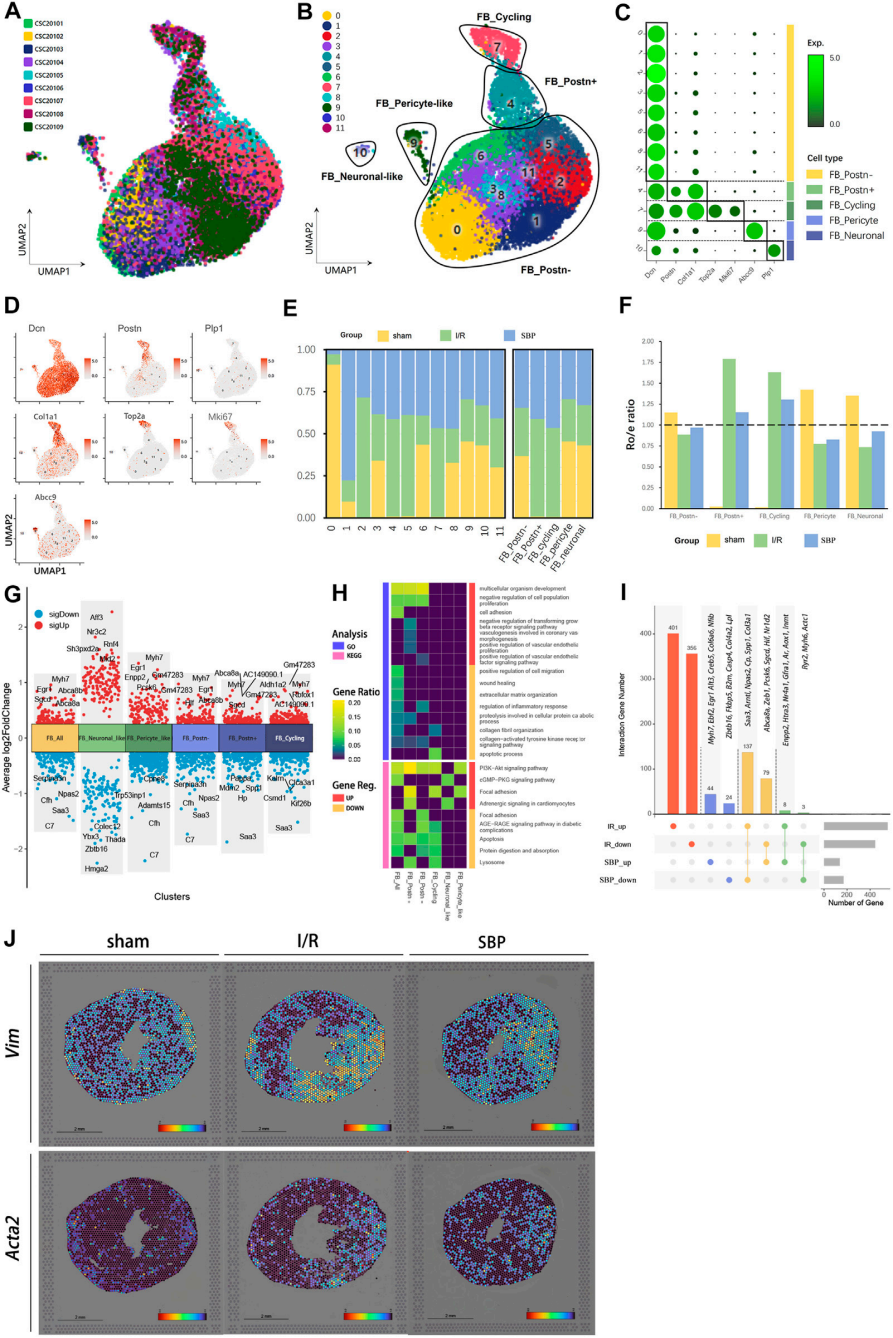
Single-cell characterization of fibroblasts in each group. **(A)** UMAP plot of fibroblasts distribution from pooled sNuc-seq data of each group tissues. **(B)** UMAP plot of fibroblast populations with different treatments from three groups. Plot is color coded by 12 clusters (0–11). **(C)** Dot plot showing fold-change (green to black) of marker genes associated with cell types and clusters. **(D)** UMAP Plot Shows marker genes. **(E)** Proportion and cell numbers of cell types and clusters relative to total number of fibroblasts in each group. **(F)** R_o/e_ shows tissue preference of each cell type in each group. **(G)** Differential gene expression analysis showing up and downregulated genes across four clusters and all fibroblasts (Y-axis: Average log2FoldChange, red: upregulated genes, blue: downregulated genes). **(H)** Heatmap plot shows the functional enrichment analysis of GO and KEGG on differential genes across each cluster and whole cells (SBP vs. I/R). **(I)** Upset plot describes different roles and target genes of SBP in the regulation of fibroblasts after myocardial I/R injury. **(J)** Spatial transcriptome data predicted the spatial location of fibroblasts (*Vim*) and myofibroblasts (*Acta2*) on heart sections.

Fibrosis is a common feature of cardiac pathological remodeling after injury. Fibroblasts proliferate and differentiate into myofibroblasts after myocardial injury, express smooth muscle actin, secrete collagen, and participate in tissue repair (Souders et al., 2009). In our study, approximately 1/4 of the cells were fibroblasts (Figure 2H). Based on marker gene characterization, we classified fibroblasts into 12 clusters and 5 cell types: postn^+^ fibroblasts (FB_Postn^+^), postn^−^ fibroblasts (FB_Postn^−^), postn^+^ cycling fibroblasts (FB_Cycling), pericyte-like fibroblasts (FB_Pericyte), and Neuronal-like fibroblasts (FB_Neuronal) (Figures 5A–D). We discovered almost no expression of *Postn* in the sham group, whereas some cells showed Postn characteristics in the I/R group (Figure 5E). *Postn* encodes a secreted extracellular matrix protein that plays a vital role in tissue development, regeneration, including wound healing, and ventricular remodeling following myocardial infarction, as well as being involved in extracellular matrix response. It has been shown that FB_Postn^+^ cells promoted scar formation and myocardial remodeling following myocardial infarction (Oka et al., 2007). In addition, the R_o/e_ plot showed a preference for Postn^+^ cells in the I/R group compared to the SBP group (Figure 5F), SBP might alleviate cardiac fibrosis after I/R injury by reducing the number of FB_Postn^+^ cells. Our differential analysis of total fibroblasts and fibroblast subgroups revealed that SBP is mainly associated with cardiac remodeling (*Erg1*, *Zbtb16*, *Myh7*, *Nr3c2*, *Serpina3n*, *Rnf4*, *Sgcd*, *Rbfox1*, *Spp1*), cell migration and proliferation (*Pappa*, *Mid2*, *Colec12*, *Kalrn*, *Csmd1*, *Kif26b*), cell death (*Enpp2*, *Mdm2*), cell contraction (*C7*), and cardiac development (*Aldh1a2*, *Adamts15*) (Figure 5G). Enrichment analysis of DEGs revealed that SBP negatively regulated cell proliferation and the TGF-β signaling pathway, and significantly upregulating the PI3K-Akt signaling pathway (Figure 5H). SBP regulated a total of 295 genes, of which 216 were reversed and 11 were further modulated. Additionally, 757 genes were unaffected by SBP, while 68 genes remained independent of the disease state (Figure 5I; Supplementary Table S5). We labeled myofibroblasts and fibroblasts with *Acta2* and *Vim*, respectively to verify our results, and discovered that *Acta2* expression was higher in the infarct and border areas of the I/R group (Figure 5J). These results indicated that fibroblasts were activated after I/R, and SBP inhibited fibroblasts activation and proliferation.

### 3.7 Effect of SBP on macrophage during I/R

**FIGURE 6:**
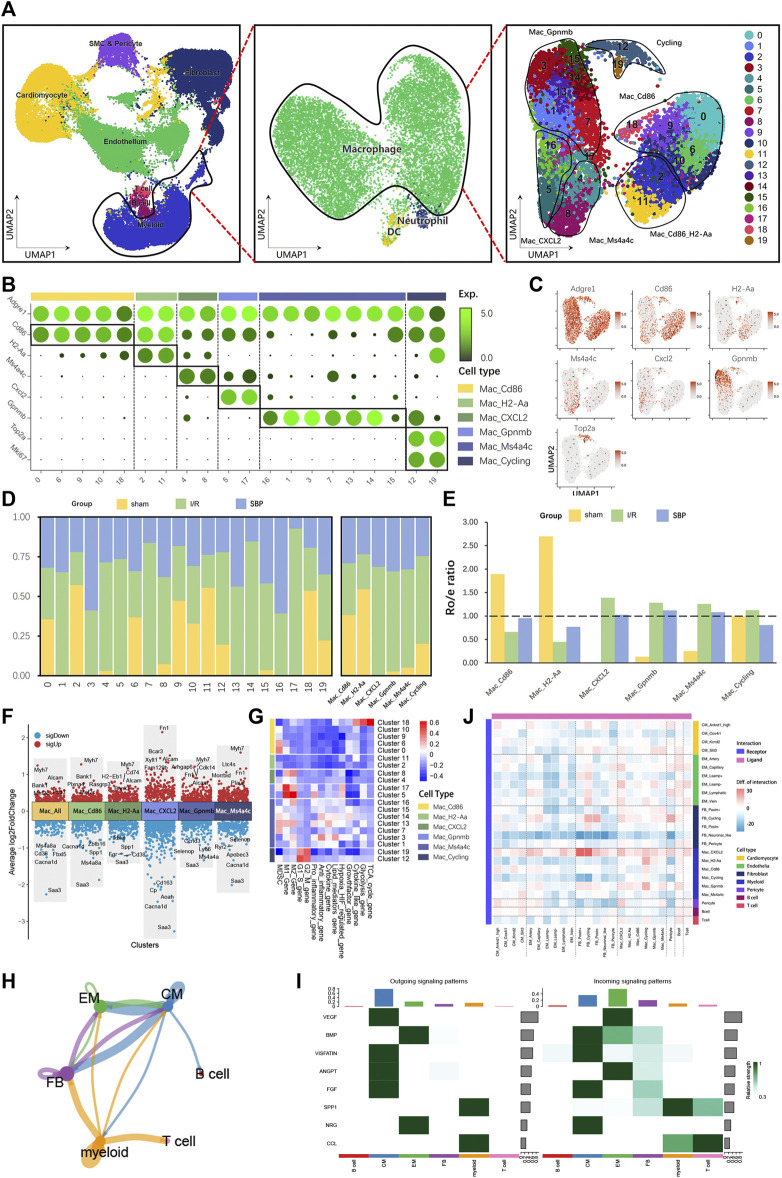
Single-cell characterization of macrophages in each group. **(A)** UMAP plot of macrophage populations with different treatments from three groups. Macrophages are extracted from myeloid cells. Plot is color coded by 20 clusters (0–19). **(B)** Dot plot showing fold-change (green to black) of marker genes associated with cell types and clusters. **(C)** UMAP Plot Shows marker genes. **(D)** Proportion and cell numbers of cell types and clusters relative to total number of macrophages in each group. **(E)** R_o/e_ shows tissue preference of each cell type in each group. **(F)** Differential gene expression analysis showing up and downregulated genes across four clusters and all macrophages (Y-axis: Average log2FoldChange, red: upregulated genes, blue: downregulated genes). **(G)** Heatmap plot showing the major functional enrichment of macrophage subgroups. **(H)** Cell-aggregated communication network: arrows represent the direction of action, colors represent the source (blue: cardiomyocytes, green: endothelial cells, purple: fibroblasts, yellow: myeloid cells), A to A autocrine, A to B paracrine, the size of the points represents the number of cells, and the thickness of the lines represents the communication intensity. **(I)** Heatmap showing the contribution of signaling pathways to cell types in terms of outgoing or incoming signaling. The color bar (right) represents the relative signaling strength of a signaling pathway across cell types (values are row-scaled). The colored bar plot (top) shows the total signaling strength of a cell types by summarizing all signaling pathways displayed in the heatmap. The right grey bar plot shows the total signaling strength of a signaling pathway by summarizing all cell types displayed in the heatmap. The y-axis represents the signaling pathway. **(J)** Heatmap plot showing the intracellular interactions for all clusters between SBP and I/R group. The difference is the number of interaction subtracted from SBP and I/R group, with red indicating enhanced interactions and blue indicating diminished interactions (Pink: As ligand, Blue-purple: As receptor).

Researchers have discovered that immune cell regulation in the heart is a highly complex network (Litviňuková et al., 2020), in which recruiting circulating immune cells are involved in cardiac remodeling, inflammatory reactions, self-repair, activating immune responses, and cell-cell interactions. Macrophages are crucial in maintaining myocardial homeostasis under normal conditions and tissue repair after injury (Ma et al., 2018). We found many highly variable genes in macrophages; therefore, we classified macrophages into 20 clusters (Figure 6A), demonstrating that macrophages are more heterogeneous. Subsequently, we merged all six clusters into six subgroups: Mac_Cd86, Mac_H2-Aa, Mac_CXCL2, Mac_Gpnmb, Mac_Ms4a4c, and Mac_Cycling (Figures 6B, C). All I/R-dominant macrophage subpopulations showed a decrease because of the effect of SBP (Figures 6D, E). Our differential analysis of total macrophages and macrophage subgroups revealed that SBP is mainly associated with polarization (*Cd163*, *Mef2c*, *Xylt1*), immune regulation (*Bank1*, *Fn1*, *Ryr2*, *Plxna4*, *Selenop*, *Plaur*), cardiac remodeling (*Cd74*, *Fam129b*, *Myh7*, *Morrbid*), cell migration and proliferation (*Fgr*, *Rasgrp3*, *Bcar3*, *Cdk14*, *Ccnd3*), inflammation (*Zbtb16*, *Ikbke*, *Saa3*, *Cd38*), and cell death (*Fbxl5*, *Ly86*) (Figure 6F). Functional enrichment showed that sham-dominant Mac_Cd86 was partially derived from peripheral monocytes, and many anti-inflammatory dominant M2 macrophages and pro-inflammatory-dominant M1 macrophages appeared after 24 h of reperfusion. However, M1 and M2 macrophages were mixed in different subpopulations, which were more difficult to distinguish using a simple marker (Figure 6G). We analyzed the cell-cell communication network to investigate the potential role of SBP in I/R. We discovered that myeloid cells were the most active cell type in the entire network and were involved in all cellular biological processes through autocrine and paracrine signaling. Fibroblasts, endothelial cells, and cardiomyocytes formed a bidirectional closed-loop; hence, neogenesis of endothelial cells or the altered contractility of cardiomyocytes were independent of each other (Figure 6H). In addition, we evaluated the signaling pathways in the SBP group (Figure 6I) and observed that cardiomyocytes were the major cells of outgoing signals, while endothelial cells received incoming signals. SPP1 signals from myeloid cells have an effect on almost all cells, except B cells. This indicates that the entire cellular regulatory network is extremely complex. We compared the number of ligand-receptor pairs for each cell type and subgroup in I/R and SBP groups. The heatmap plot revealed that several cell types exhibited increased or decreased cell-cell communication with themselves (autocrine) and with other lineages (paracrine) after SBP treatment (Figure 6J), especially FB_Postn^+^ and Mac_CXCL2. Therefore, we suggest that SBP may play a role in regulating the cellular microenvironment.

## 4 Discussion

The pathophysiological mechanisms underlying I/R injury are complex, and the clinical safety of drugs for I/R requires improvement. The development and research of new drugs and their clinical application entail a prolonged process. TCM has better application prospects in treating complex diseases because it has multicomponent, multi-target, and multi-signaling pathways. SBP was processed from seven medicinal materials. SBP is a traditional Chinese medicine composed of seven herbal ingredients, including artificial musk, ginseng extract, artificial bezoar, cinnamon, mosla herb, toad venom, and borneol. Currently, over 110 compounds have been detected in SBP, with 11 active ingredients (Lv et al., 2017) and four blood components (Chang et al., 2014) quantified. Multiple chemical components, such as ginsenosides Rb2 and Rg3, cinnamaldehyde, and borneol, have been proven to have cardioprotective effects. Over 40 years of clinical application has shown that it is safe and effective and is recommended for use in CAD and ACS by the clinical application of the Chinese Coronary Heart Disease Association guidelines. Therefore, we selected the anterior descending branch ligation and recanalization model to simulate patients with percutaneous coronary intervention recanalization after MI and pre-treated the mice with SBP to explore the role and mechanism of SBP in reducing myocardial I/R injury. SNuc-seq and ST-seq provide an important basis for further studies on SBP and its novel therapeutic targets.

Cardiomyocytes are important components of the heart and are responsible for contraction and pumping of blood. Myocardial I/R result in significant myocardial cell death and a deterioration in cardiac function. Therefore, reducing the rate of cardiomyocyte death is crucial. We examined whether SBP could attenuate I/R-induced damage to cardiomyocytes. Unfortunately, no significant difference in the percentage of cardiomyocytes was observed between the SBP and I/R groups, suggesting that SBP did not reduce cardiomyocyte death in the early stages. The infarct size demarcated by TTC staining manifested a declining trend when comparing the SBP group with the I/R group (I/R: 24.29% ± 3.48%; SBP: 21.60% ± 3.83%). Meanwhile, ST-seq showed that SBP reduced the area of infarction in the infarction size (SBP: 15% vs. I/R: 27%). Albeit this intriguing tendency, a statistically significant distinction between the two cohorts was absent. The underlying rationale for this phenomenon could be ascribed to the suboptimal sensitivity of TTC staining in infarction size detecting (Ito et al., 1997). Some clinical studies (Qin et al., 2021; Wei et al., 2021) have found that SBP increased LVEF and decreases BNP levels. However, our study results were not entirely consistent with this finding. In our study, the pre-treatment of SBP in mice resulted in further upregulation of the BNP encoding gene after I/R for 24 h. We believed that the reason for this difference might be due to differences in transcription and protein levels. In addition, differences in drug dosage, observation time, treatment strategies, and mechanisms, such as acute I/R and long-term myocardial infarction, may also be factors. Differential gene analysis showed that SBP upregulates the expression of genes related to myocardial contraction and hypertrophy, which was confirmed in UCG and pathology. We know that *Myh7*, *Actc1*, *Tpm1*, and *Pdlim3* are known hypertrophic regulatory genes (Cirino and Ho, 1993), SBP could upregulate the expression of these genes. Interestingly, *Mhrt* is a newly discovered long non-coding RNA which could negatively regulate the expression of *Myh7* (Han et al., 2014). We found that SBP downregulated the expression of *Mhrt* and upregulated the expression of *Myh7*. These results were both consistent and suggested that SBP might promote compensatory hypertrophy of cardiomyocytes and increase cardiac output and contraction to alleviate I/R damage in the early stage through the regulation of hypertrophic-related gene expression, which is beneficial (Burchfield et al., 2013). Furthermore, we found that SBP significantly upregulated the expression of *Nppb* in the infarct and border areas, which we know that BNP was released in large amounts under pressure load and protected the heart by diuresis, vasodilation, and inhibition of aldosterone secretion (Kuwahara, 2021). This phenomenon may potentially be explained by the significant regulation of SBP on myocardial energy metabolism, amino acid metabolism, and lipid metabolism, as observed in proteomics and metabolomics studies (Xiang et al., 2012; Song et al., 2022). In addition, we identified I/R-independent targets (non-DEGs between I/R and sham) in each cell type. These genes may help us deepen our understanding of SBP, which we will investigate in the future. Summarily, the mechanism of SBP in cardiomyocytes may not be to reduce cardiomyocyte mortality in the early stages but to promote compensatory hypertrophy of cardiomyocytes and increase cardiac output and contraction to alleviate I/R damage.

Angiogenesis is an important protective effect during I/R, which induces capillary network formation and restores the equilibrium of cellular supply and demand for blood (Sun et al., 2018). Previous studies (Huang et al., 2017; Yuan et al., 2018) have demonstrated that SBP can promote angiogenesis after MI by promoting endothelial cell proliferation through *VEGF* upregulation. *In vitro* studies have provided additional insights into the mechanisms underlying the beneficial effects of certain active components of SBP, including ginsenoside Rb2, ginsenoside Rg3, and cinnamaldehyde. These components have been shown to promote endothelial cell proliferation and tube formation, thereby facilitating angiogenesis, an effect that is mediated by the PI3K/Akt and MAPK/ERK1/2 signaling pathways (Yuan et al., 2018; Choi et al., 2021). We discovered that SBP altered the percentage of endocardial cells and capillaries but not arterial, venous, or lymphatic endothelial cells. The endocardium is a natural biological barrier between the circulating blood of the ventricles and cardiomyocytes, including endocardial and microvascular endothelial cells. The endocardium is essential for cardiac embryonic development, cardiac contraction, rhythm, and remodeling (Brutsaert, 2003;Smiljić et al., 2010). Endocardial injury reduces endothelial-mediated NO release and cardiomyocyte sensitivity to Ca^2+^ and contractility (Shen et al., 2013). GO analysis revealed that SBP downregulates NO-mediated signal transduction. Therefore, we hypothesized that SBP could improve cardiomyocyte contractility by reducing endocardial cell injury and promoting NO release. Furthermore, DEGs enrichment analysis revealed that endocardial cells were related to hypertrophy and cell migration at the gene transcription level, we suggested that hypertrophy might be related to the secretion of large amounts of growth factors and extracellular matrix proteins by endocardial cells under stressful conditions, thereby regulating cardiac contractility and cardiomyocyte remodeling through paracrine (Frangogiannis, 2012) (Segers et al., 2018). Furthermore, the endocardial marker gene *Npr3* was significantly upregulated in the SBP group. Spatial mapping revealed that endocardial cells are expressed in the infarct and border areas, which was absent in the I/R and sham groups. However, the contribution of endocardial cells to angiogenesis is currently controversial (Tang et al., 2018); they found a minimal contribution of endocardial cells to angiogenesis after MI using *Npr3*-CreER genetic lineage tracing, suggesting that ischemia and hypoxia did not promote endocardial proliferation and internalization, which is similar to our findings. However, the abnormal expression of *Npr3* after SBP pre-treatment piqued our interest. We observed a reduction in capillary endothelial cell numbers, which was unexpected. However, research indicates that endothelial cells enter a transient endothelial-mesenchymal activation state following myocardial infarction, losing expression of endothelial cell marker genes and leading to a reduction in capillary endothelial cell numbers within 1 week (Tombor et al., 2021). Thus, we hypothesize that SBP may promote capillary endothelial cell mesenchymal transition in the early stage of I/R, thereby promoting vascular network regeneration. Hence, the role of SBP in endocardial cells is of great research value and may be a potential target for cardiac angiogenesis. We are the first to report the specific effects of SBP on the endocardium.

Cardiac remodeling is an unavoidable pathological process after a MI, where the loss of functional cardiomyocytes causes a high state of stress in the remaining cardiomyocytes and elevated intracardiac pressure. This injury activates and proliferates cardiac fibroblasts, some of which produce excessive extracellular matrix components that impair myocardial structure and function (Fan et al., 2012; Ali et al., 2014), causing myocardial hypertrophy and fibrosis (Dong et al., 2018). Therefore, inhibiting cardiac remodeling is key to I/R. Some researchers have suggested that SBP might lower TGF-β1 levels, reduce mitochondrial swelling, improve systolic and diastolic functions, and delay or reverse cardiac fibrosis progression (Liu et al., 2012). In our study, fibroblasts were activated and proliferated within 24 h after I/R, and SBP restricted this cell fraction. We analyzed the characteristics of FB_Cycling cells to determine the differences in proliferating state cells and found that SBP negatively regulated VEGF production and the TGFR-β signaling pathway, which are essential for fibroblast activation and proliferation. In addition, SBP downregulated IL-1-induced inflammatory signaling and controlled IL1/fibroblast paracrine signaling after ischemia, which regulate post-MI remodeling and improve cardiac function in animal models (Bujak et al., 2008; Bageghni et al., 2019). In addition, fibroblast activation is an important factor contributing to fibrosis, and MI triggers the activation of resident fibroblasts, causing them to transform from a resting state to highly active myofibroblasts that migrate to injured areas and proliferate, secrete ECM, and shrink scar tissue (Herum et al., 2017). We mapped the location of fibroblasts (*Vim*) and myofibroblasts (*Acta2*) using ST-seq and found that myofibroblasts were present in the infarcted area after I/R. In contrast, the expression and distribution of myofibroblasts were significantly reduced in the SBP group, indicating that SBP partly prevented fibroblast activation. Moreover, SBP negatively regulated the TGFR-B and HIF signaling pathways, which might be related to fibroblast-to-myofibroblast conversion inhibition by the TGF-β1/HIF-1a/aerobic glycolytic signaling cascade (Zou et al., 2022). Moreover, we found that the signal pathways were mostly related to vascular functions, Changes in the relative proportions of different fibroblast subpopulations may have significant effects on the entire vascular wall. Studies have shown that under hypoxic conditions, exogenous fibroblasts may serve as a key regulator of vascular function and structure. Fibroblasts can complete the transition to become vascular wall myofibroblasts, while *in vitro* studies have confirmed that the stability of fibroblast phenotype is related to the signaling pathways that induce proliferation. Their unique ability to proliferate, differentiate, and migrate allows for a rapid response to hypoxic stress, thereby regulating their function to adapt to local vascular demands (Stenmark et al., 2002). Interestingly, SBP inhibited the activation and proliferation of fibroblasts. Nevertheless, Figure 2I showed that the fibroblasts in the SBP group was increased, and we found that the number of fibroblasts containing other cell characteristics was greater in the SBP group than in the I/R group, suggesting that some of the fibroblasts might be in the transition state. However, the mechanisms were complex, so we speculated that the increase of fibroblasts might be caused by the combined effects of reduced proliferation and increased transformation. Our findings established that SBP plays an antifibrotic role in I/R injury. Fibrosis can prevent the heart from rupture and has a stabilizing effect in MI early stages. SBP inhibits fibrosis in the early stages of reperfusion; however, whether it is beneficial or detrimental remains to be investigated in the future.

Cardiac immunity is an important pathophysiological factor and target of MI, characterized by the recruitment and activation of immune cells that coordinate a highly complex immune response throughout the process from ischemia to fibrosis (Frangogiannis, 2012). However, many current clinical trials on immune regulation after MI are controversial (Panahi et al., 2018), suggesting that little is known about the complex diversity of cardiac immunity. Therefore, we investigated the role of SBP in the early stages of I/R through cell-cell communication network analysis and found that it was fascinating and complex. Damaged cardiomyocytes affected endothelial cell, fibroblast, myeloid cell, and B cell development by secreting many cytokines. The most notable of these is VEGF signaling, where cardiomyocytes release numerous VEGF signals, which in turn were absorbed by endothelial cells, affecting cardiomyocyte function through BMP and NRG signaling. In addition, SPP1 signaling from myeloid cells influences several cells such as fibroblasts, T cells, and myeloid cells. We further analyzed the communication network between cell subgroups in the I/R and SBP groups and discovered that the number of ligand-receptor pairs between FB_Postn^+^ and Mac_CXCL2 was significantly altered, implying that SBP may be involved in cardiac fibrosis and scar healing through the cell-chat between Mac_CXCL2 and FB_Postn^+^. SBP also increased the number of interactions between endothelial cells and macrophages, demonstrating that it can promote angiogenesis by activating macrophages to regulate endothelial cell function and signal transduction pathways (Zhang J et al., 2020). Thus, SBP is crucial in macrophage regulation. However, our study focused on the early phases of I/R, and macrophages are predominantly pro-inflammatory in the early phases of post-ischemia, although they demonstrate distinct anti-inflammatory effects in the later phases and are involved in cardiac injury repair. Therefore, further studies on macrophages are required. Our study showed that SBP modulates the ligand-receptor pair relationship in cells and induces macrophages to participate in pathological changes in I/R.

This study had two limitations. Firstly, the small sample size resulted in a limited capture of smooth muscle cells. Despite our observation that SBP affects the quantity of smooth muscle cell, we regret that further analysis would increase the risk of result bias. Therefore, we refrained from delving into a discussion of smooth muscle cells. Secondly, due to the limitation of our research conditions, we used sNuc-seq to study the effects of SBP on the heart, this may easy to lose part of immune cells, making the function of immune cells unable to be fully explained. Despite these limitations, our study preliminarily described the ecological changes of various cardiac cells after SBP intervention and elucidated the effect of SBP on I/R in mice. To the best of our knowledge, this is the first study to report the mechanisms and cell landscape of SBP at a single-cell level and spatial resolution.

## 5 Conclusion

SBP improves the early LVEF of I/R mice and has a cardioprotective effect. Through sequencing analysis, we observed that SBP can increase the gene expression of *Nppb* and *Npr3* in the infarct area of the heart. *Npr3* is related to vascular generation mediated by endocardial cells and requires further research. In addition, SBP increases the number of fibroblasts, inhibits the expression of genes related to fibroblast activation and proliferation, and increases the transformation of endothelial cells into fibroblasts. These findings will help to indicate directions for further research.

## Data Availability

The datasets presented in this study are deposited in the GEO repository, accession numbers: GSE227238 and GSE227088.
