# Supplementary material for: Single-nucleus ribonucleic acid-sequencing and spatial transcriptomics reveal the cardioprotection of Shexiang Baoxin Pill (SBP) in mice with myocardial ischemia-reperfusion injury
Source: Front Pharmacol. 2023 May 9;14:1173649. doi: 10.3389/fphar.2023.1173649 (PMC10203427; doi:10.3389/fphar.2023.1173649)
Supplement: Supplementary file 1 [file DataSheet1.pdf]

Supplementary Material

Single-nucleus Ribonucleic Acid-sequencing and Spatial Transcriptomics Reveal the Cardioprotection of Shexiang Baoxin Pill (SBP) in Mice with Myocardial Ischemia-Reperfusion Injury

Wenyong Lin[1,2], Xin Chen[3], Dongyuan Wang[1,2], Ruixia Lu[1,2], Chunling Zhang[1], Zhenchao Niu[1,2], Jie Chen[1], Xiaofen Ruan[1,2], Xiaolong Wang[1,2*]

[1] Branch of National Clinical Research Center for Chinese Medicine Cardiology, Shuguang Hospital Affiliated to Shanghai University of Traditional Chinese Medicine, Shanghai, China

[2] Cardiovascular Research Institute of Traditional Chinese Medicine, Shuguang Hospital Affiliated to Shanghai University of Traditional Chinese Medicine, Shanghai, China

[3] Shanghai Innovation Center of TCM Health Service, Shanghai University of Traditional Chinese Medicine, Shanghai, China

*** Correspondence:**
Xiaolong Wang
E-mail address: wxlqy0214@163.com

1 Supplementary data

1.1The analytical parameters for the compositional analysis of SBP.

Liquid chromatography conditions: The mobile phase was a gradient elution of 0.1% formic acid aqueous solution (C) - acetonitrile (D), with the following program: 0-1 min, 95% C-5% D; 1-6 min, 68% C-32% D; 6-12 min, 45% C-55% D; 12-19 min, 5% C-95% D; 19-22 min, 95% C-5% D. The flow rate was 0.3 mL/min, the column temperature was 45℃, and the injection volume was 3 μL.

Mass spectrometry conditions: The ion source was HESI; positive ion spray voltage: 3.5 kV, negative ion spray voltage: 2.5 kV; auxiliary gas flow rate 12 L/min, sheath gas flow rate 40 L/min, capillary temperature 330℃, auxiliary gas heating temperature 300℃, collision energy (CE) 50 eV. The detection mode was Full MS, with a Full MS resolution of 70,000 and a scan range of m/z 80-1200.

2. Supplementary Figures and Tables

2.1 Supplementary Figures

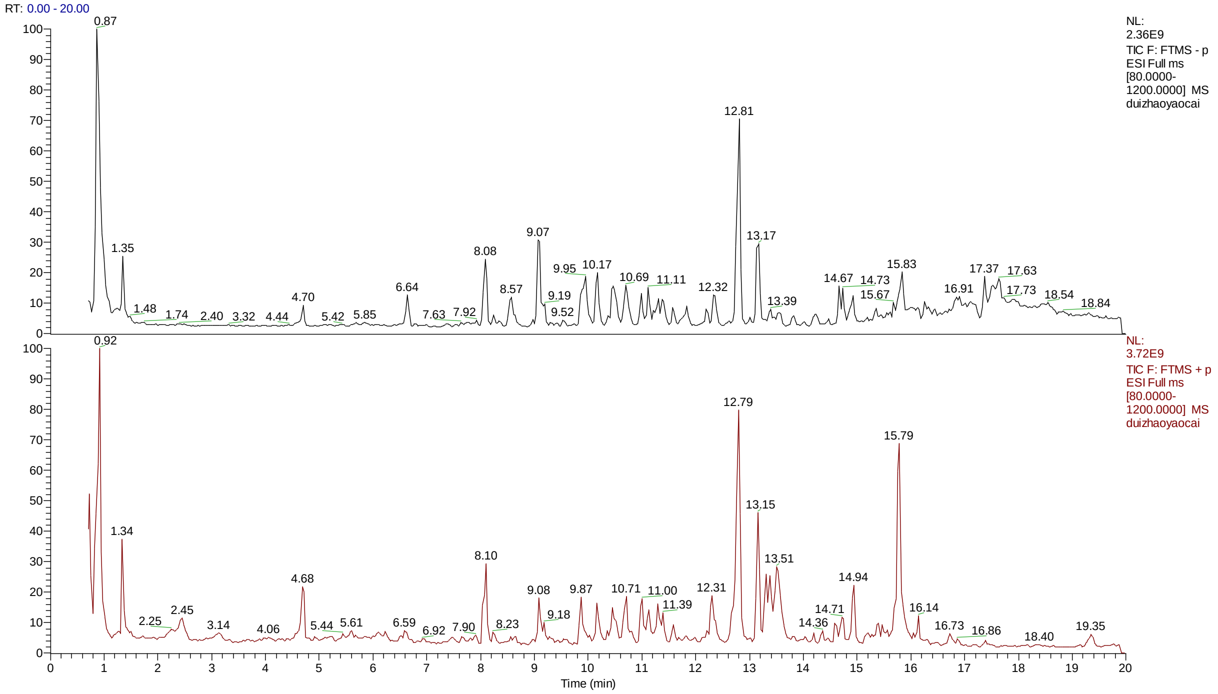

Supplementary Figure 1. Total ion chromatogramof the sample of SBP(TIC)

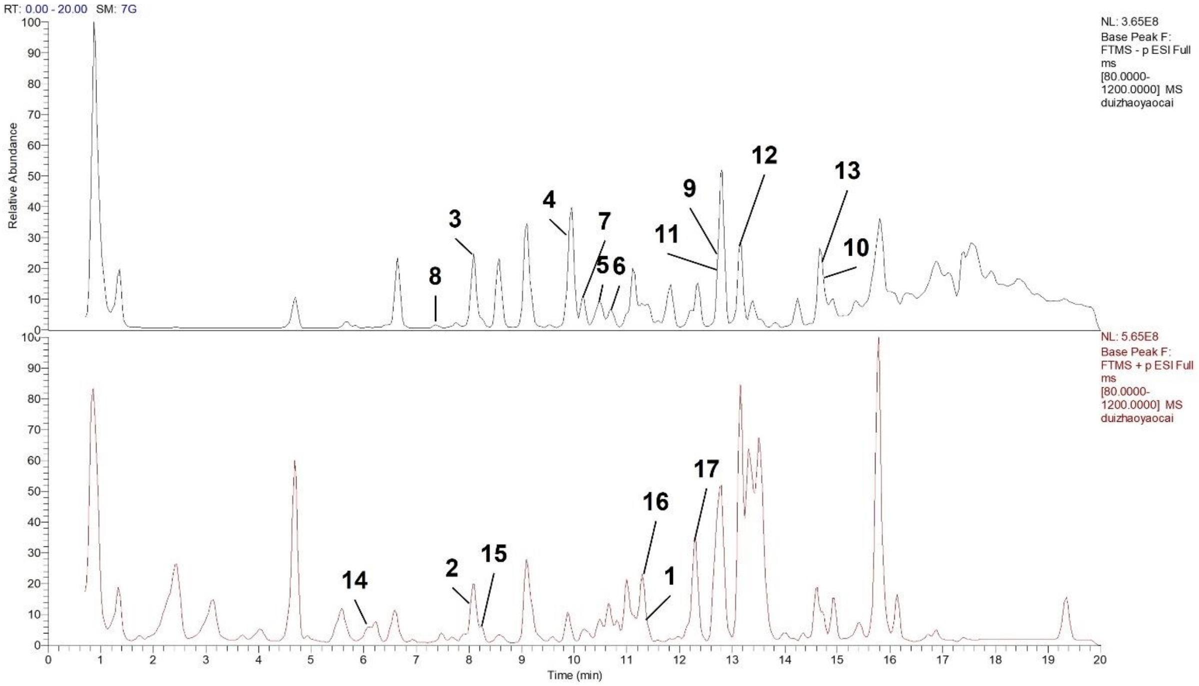

Supplementary Figure 2. Base peak chromatogram of the sample of SBP(BPC)

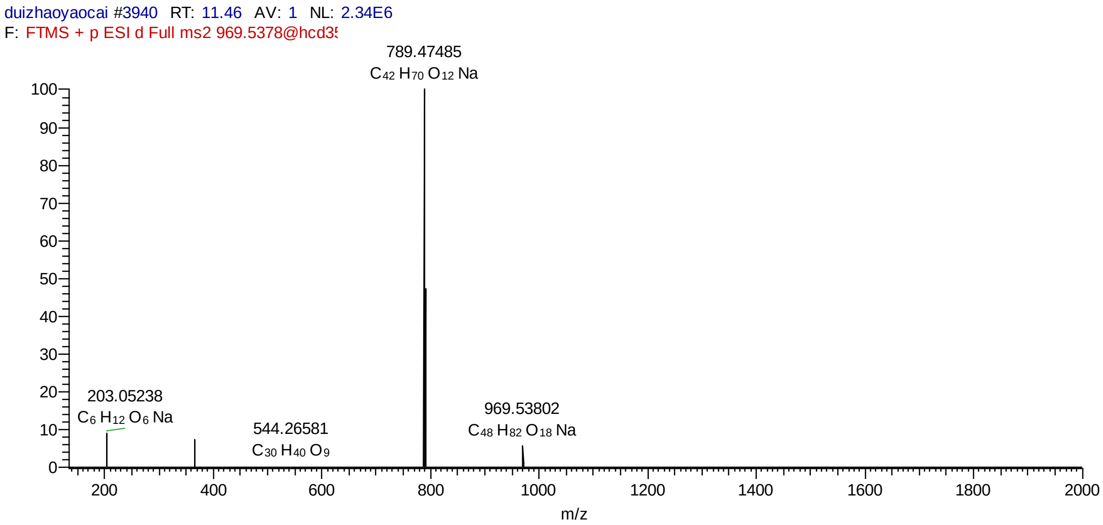

Supplementary Figure 3. MS/MS diagram for the extraction of Ginsenoside Rd and Ginsenoside Re.

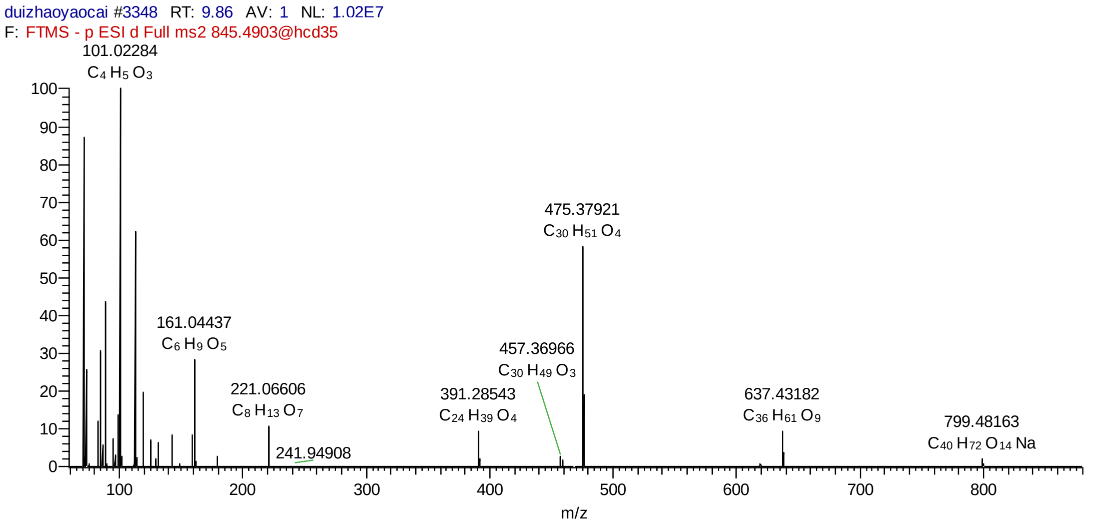

Supplementary Figure 4. MS/MS diagram for the extraction of Ginsenoside Rg1.

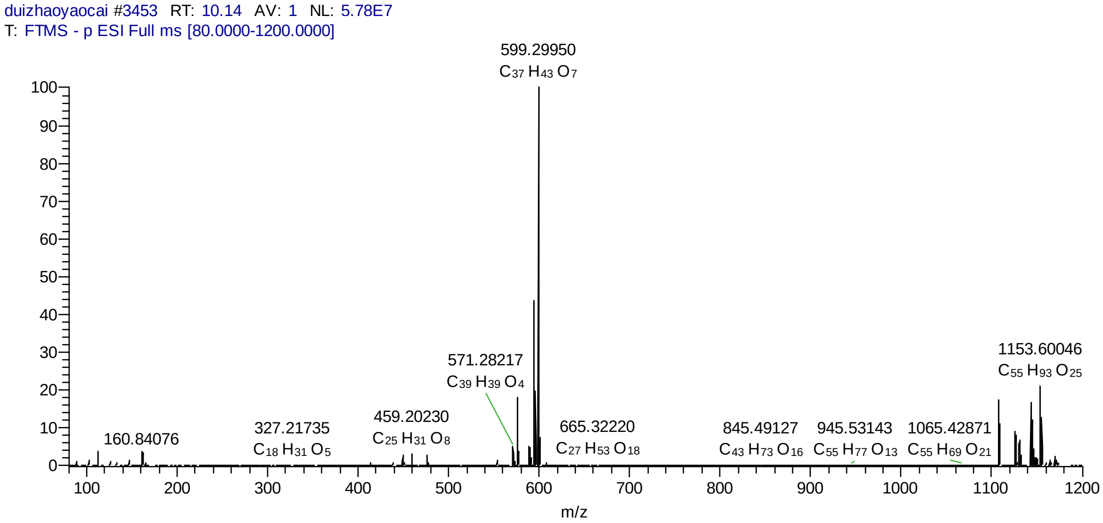

Supplementary Figure 5. MS/MS diagram for the extraction of Ginsenoside Rb1.

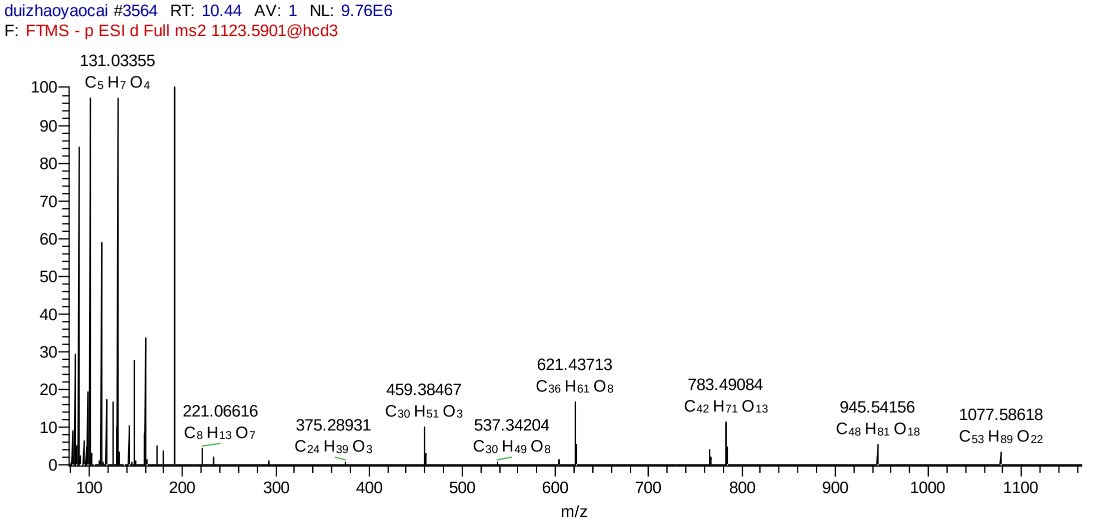

Supplementary Figure 6. MS/MS diagram for the extraction of Ginsenoside Rb2 and Ginsenoside Rb3.

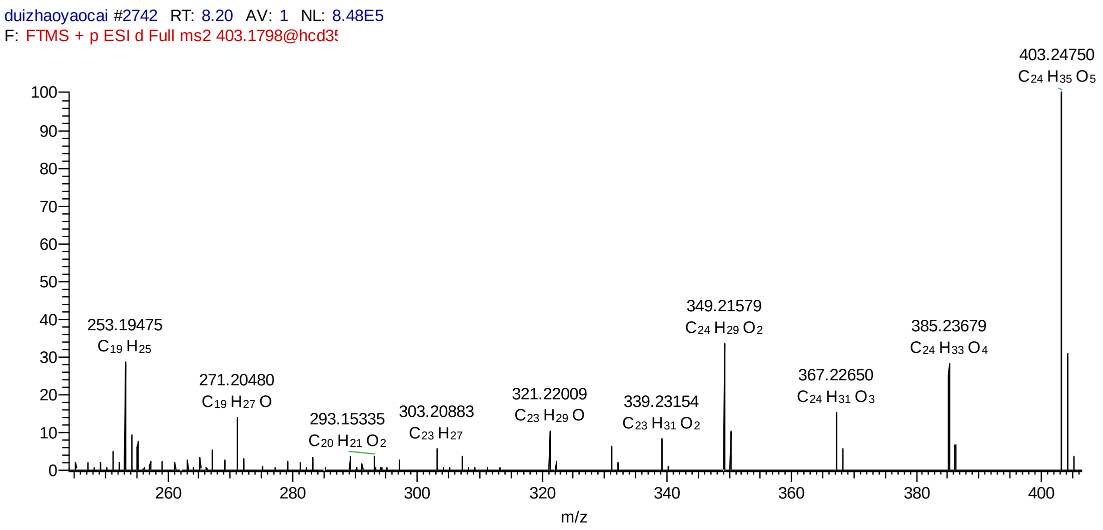

Supplementary Figure 7. MS/MS diagram for the extraction of Gamabufotalin.

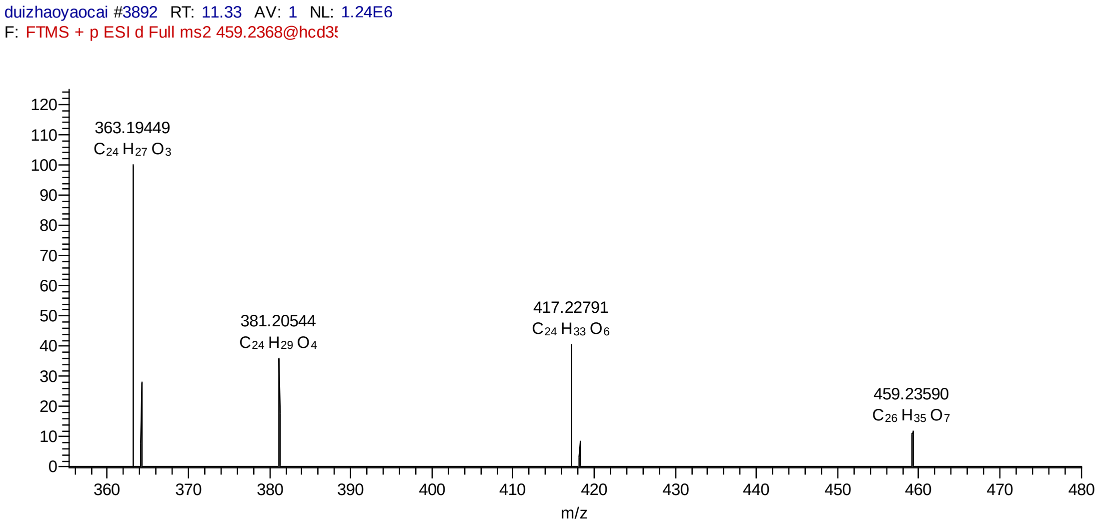

Supplementary Figure 8. MS/MS diagram for the extraction of Cinobufotalin.

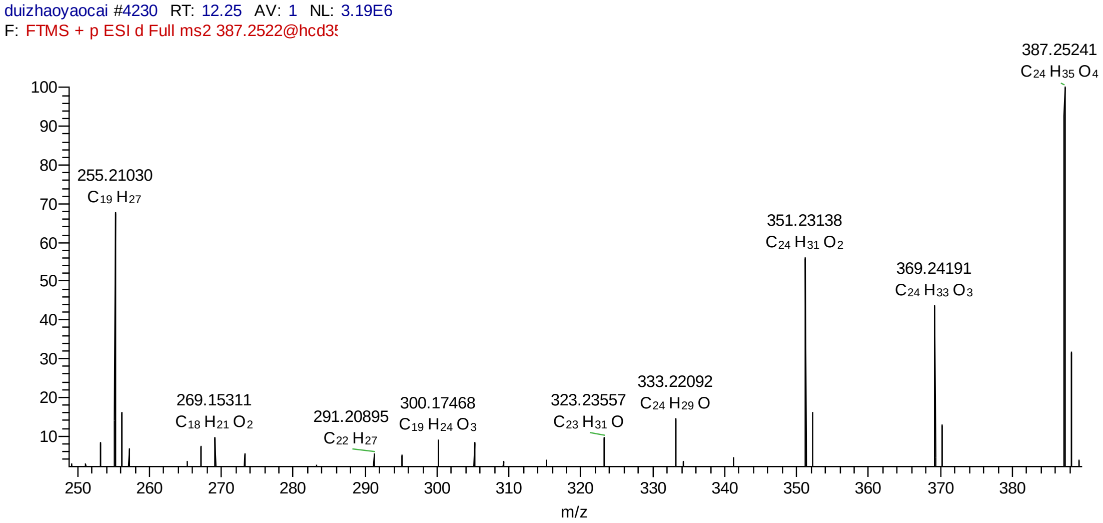

Supplementary Figure 9. MS/MS diagram for the extraction of Bufalin.

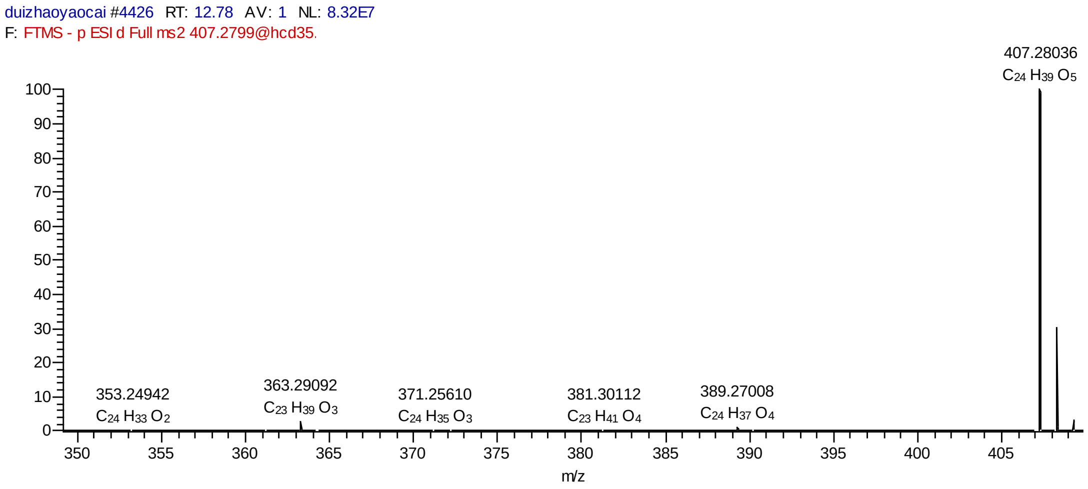

Supplementary Figure 10. MS/MS diagram for the extraction of Cholic acid.

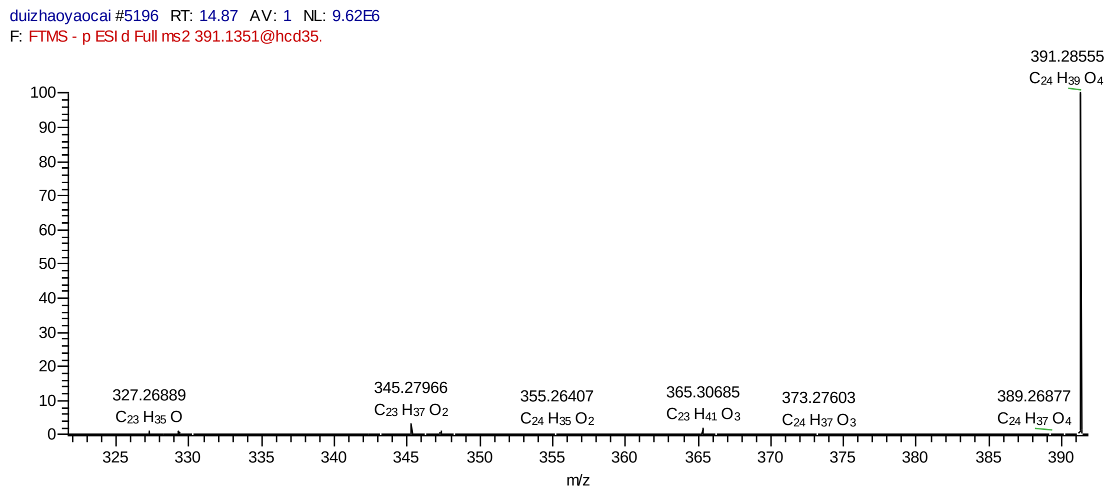

Supplementary Figure 11. MS/MS diagram for the extraction of Deoxycholic acid, Ursodeoxycholic acid, Chenodeoxycholic acid, and Hyodeoxycholic acid.

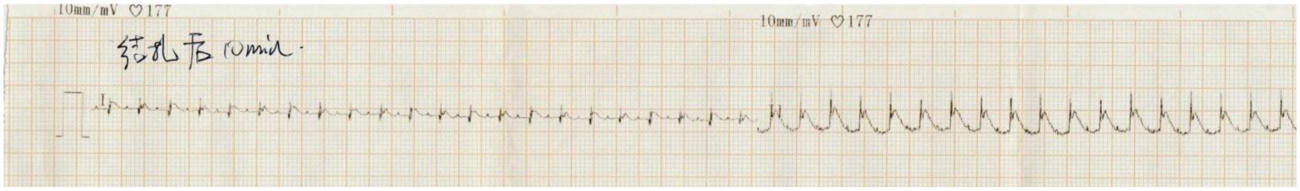

Supplementary Figure 12. The ECG taken 10 minutes after coronary artery ligation.

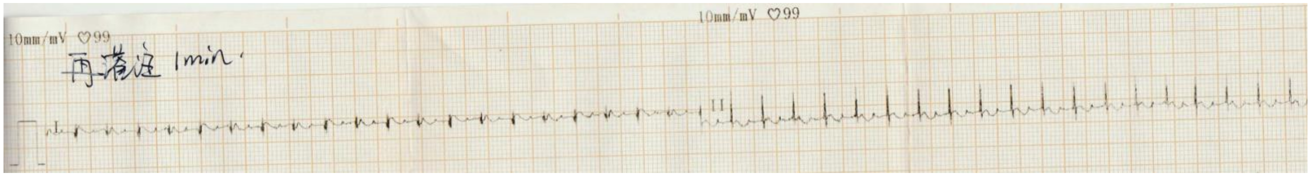

Supplementary Figure 13. The ECG taken 1 minute after coronary artery reperfusion.

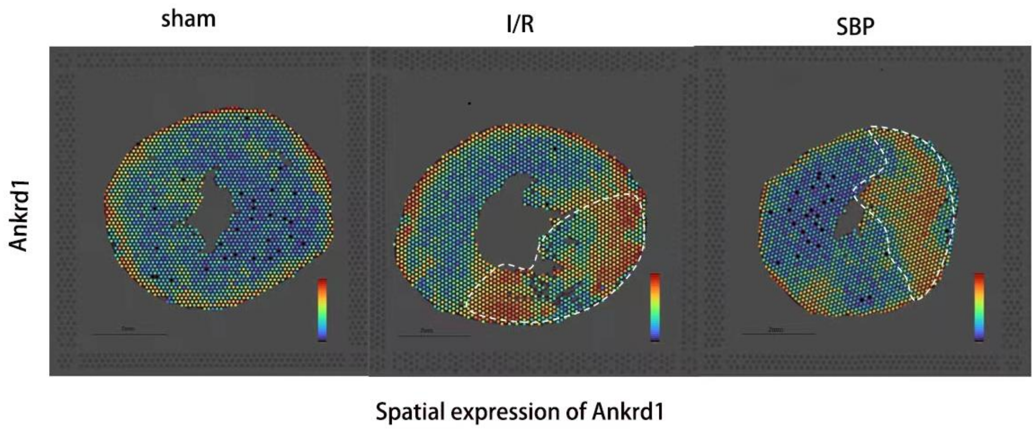

Supplementary Figure 14. Spatial transcriptome data predicted the spatial location of Ankrd1 on heart sections . Spatial transcriptome data predicted the spatial location of Ankrd1 on the cardiac section. We used Ankrd1 as a marker to distinguish infarct and non-infarct areas, with I/R infarct areas smaller than SBP (SBP: 15% vs. I/R: 27%).

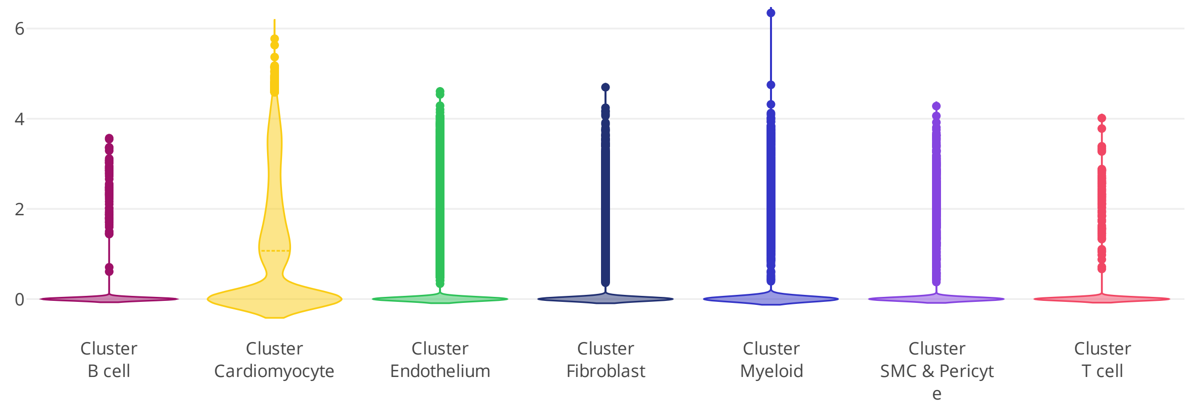

Supplementary Figure 15. The mean expression of Myh7 in B cell (0.414), Cardiomyocyte (1.174), Endothelium (0.491), Fibroblast (0.413), Myeloid (0.450), SMC & Pericyte (0.445), and T cell (0.349).

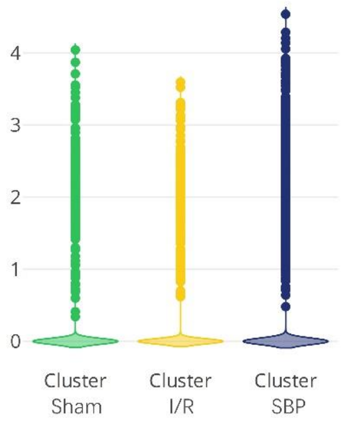

Supplementary Figure 15. The mean expression of Myh7 in three groups (Sham: 0.154 vs I/R: 0.120 vs SBP: 0.513) of endothelial cells.

2.2 Supplementary Tables

Supplementary Table 1. Qualitative results of 17 components in SBP

Peak No.	Compound	molecular formula	M±(m/z)	Rt	Fragments ions (m/z)
1	Ginsenoside Rd	$C_{48}H_{82}O_{18}$	969.53934[M+Na]+	11.39	789.47485,
2	Ginsenoside Re	$C_{48}H_{82}O_{18}$	969.53934[M+Na]+	8.04	789.47485,
3	Ginsenoside Rg1	$C_{42}H_{72}O_{14}$	845.48931[M+HCOO]-	8.08	637.43182,475.37921
4	Ginsenoside Rf	$C_{42}H_{72}O_{14}$	845.48931[M+HCOO]-	9.85	637.43182,475.37921
5	Ginsenoside Rb2	$C_{53}H_{90}O_{22}$	1123.58948[M+HCOO]-	10.43	945.54156,783.49084,621.43713, 459.38467
6	Ginsenoside Rb3	$C_{53}H_{90}O_{22}$	1123.58948[M+HCOO]-	10.69	945.54156,783.49084,621.43713, 459.38467
7	Ginsenoside Rb1	$C_{54}H_{92}O_{23}$	1107.59457[M-H]-	10.17	945.53143,621.31171,459.20230
8	Cinnamic acid	$C_{9}H_{8}O_{2}$	147.04406[M-H]-	7.36	
9	Cholic acid	$C_{24}H_{40}O_{5}$	407.2792[M-H]-	12.78	389.27008,371.25610,353.24942
10	Deoxycholic acid	$C_{24}H_{40}O_{4}$	391.28429[M-H]-	14.84	

11	Ursodeoxycholic acid	C24H40O4	391.28429[M-H]-	12.55	373.27603,355.26407,327.26889
12	Hyodeoxycholic acid	C24H40O4	391.28429[M-H]-	13.17	373,355,328
13	Chenodeoxycholic acid	C24H40O4	391.28429[M-H]-	14.73	
14	Cinnamic aldehyde	C9H8O	133.06479[M+H]+	6.1	
15	Gamabufalin	C24H34O5	403.2479[M+H]+	8.23	385.23679,367.22650,349.21579, 253.194758
16	Cinobufagin	C26H34O7	459.23773[M+H]+	11.29	417.22791,381.20554,363.19449
17	Bufalin	C24H34O4	387.25299[M+H]+	12.31	36924191,351.23138,333.22092, 305.22653,255.21030

Supplementary Table 2. Quality control results of sNuc-seq.

Sample	CSC20101	CSC20102	CSC20103	CSC20104	CSC20105	CSC20106	CSC20107	CSC20108	CSC20109
before_filtering_total_reads	665986854	769776542	653899990	754877490	713499600	730060644	699149168	774527380	722051630
before_filtering_total_bases	99898028100	1.15466E+11	98084998500	1.13232E+11	1.07025E+11	1.09509E+11	1.04872E+11	1.16179E+11	1.08308E+11
before_filtering_q20_bases	81208125981	93653494538	79696479917	89816436588	84787183725	87011954386	83397122048	91991449550	86231101272
before_filtering_q30_bases	70742722736	81390786037	69094891359	76741057783	72118457551	74434310549	71329631396	77994991723	73617328044
before_filtering_q20_rate	0.8129102	0.811088149	0.812524659	0.793209828	0.792218933	0.794563713	0.795224881	0.791807167	0.796167455
before_filtering_q30_rate	0.708149341	0.704886692	0.70443893	0.677735207	0.673847213	0.679708927	0.680156536	0.671334061	0.679705116

before_filtering_gc_content	0.353098	0.351926	0.352008	0.375601	0.388381	0.378955	0.379662	0.380862	0.38247
after_filtering_total_reads	665446582	769133416	653412676	754344040	713006230	729609418	698638056	773921368	721495490
after_filtering_total_bases	59193431475	68418013475	58129017761	67107669103	63435228362	64912578936	62154343700	68856832544	64189657900
after_filtering_q20_bases	57573974254	66458757435	56289400521	64283442723	60510519070	62357372312	59621276913	65362530349	61440328214
after_filtering_q30_bases	54937398240	63285329803	53331794100	60382194776	56493681083	58630765986	56039749541	60724119782	57550832936
after_filtering_q20_rate	0.972641268	0.971363447	0.968352859	0.957914998	0.953894557	0.960636187	0.959245539	0.949252644	0.95716865
after_filtering_q30_rate	0.928099569	0.924980522	0.917472824	0.899780839	0.890572676	0.903226569	0.901622416	0.881889531	0.896574851
after_filtering_gc_content	0.438676	0.43819	0.437375	0.431525	0.446567	0.435808	0.437294	0.438868	0.439948
ReadsFilter%	99.91887648	99.9164529	99.92547576	99.9293329	99.9308521	99.93819335	99.92689514	99.92175719	99.92297781
BaseFilter%	59.25385376	59.253571	59.26392277	59.26583672	59.27144492	59.27596972	59.26665014	59.26782734	59.26599081
low_quality_reads	52	80	52	7270	6682	6856	6600	6948	6770
too_many_N_reads	44142	50476	43144	24006	22998	23460	22490	25064	22924
too_short_reads	496078	592570	444118	502174	463690	420910	482022	574000	526446
too_long_reads	0	0	0	0	0	0	0	0	0

Supplementary Table 3. The list of disease-independent genes in cardiomyocyte.

	SBP_upregulate	SBP_downregulate
Gene Symbol	Tpm1, Cacna1c, Ldb3, Actn2, Jph2, Pkig, Cdh2, Gm30624, Mlip, Mylk3, Pde4d, Rftn1, Atp8a1, Sik3, Idh2, Cap2, Mtus2, 5430431A17Rik, Smyd1, Mical2, Hk1, Ctnnal1, Ldlrad4, Dstn	Zbtb16, Fkbp5, Pik3r1, Ppip5k2, Ryr2, Ddx5, Tead1, Rgs7, Arrdc2, Gm40841, Gpcpd1, Ptgds, Nt5e, Herpud1, Klhl38, Tnik, B2m, Agtpbp1, Ptpn11, Vldlr, Tob2, Eef2k, Ogdhl, Myoz2, Ppp1r3a, Slc25a33, Snrnp70, Atrnl1, Pid1, Dysf, H2-K1, Camk1d, Pfkfb1, Bsg, Epha4
count	24	35

Supplementary Table 4. The list of disease-independent genes in endothelium cell.

	SBP_upregulate	SBP_downregulate
Gene Symbol	Myh7, Ppp3ca, Airn, AC149090.1, H19, Ebf1, Car8, Slc6a6, Sema6a, Tmem108, Gm47283, Agap1, Plxna4, Foxp1, Ncam1, Cgnl1, Rasgef1b, Ifitm10, Greb1l, Pgm5, Csgalnact1, Zfp608, Rbms3, Ttc28, Upp2, Rassf9, Fgf14, Astn2, Epha7, Emcn, Apba1, Zfp521, Tspan2, Nrxn3, Cdc14a, Acvr1, Npr3, Kank1, Bace2, Pir, Bmp6, Ntn1, Megf9, Sox5, Itgbl1, Rasgrf2, Nuak1, Sobp, Adgrg6	Zbtb16, B2m, Plxna2, Lpl, Acyp2, Klf13, Arhgap15, Tnik, Calm1, Klf9, Birc3, Slc9a9, Sod2, Fmnl2, Rin3, Hecw2, Pik3ip1, Ptp4a2, Ddx60, Nebl, Igfbp7, Birc2, Fam168a, Timp3, Vcam1
count	49	25

Supplementary Table 5. The list of disease-independent genes in fibroblast.

	SBP_upregulate	SBP_downregulate
Gene Symbol	Myh7, Ebf2, Egr1, Ebf1, Dlc1, Bicc1, Ppl, Creb5, AC149090.1, Akt3, Gxylt2, Col6a6, Nfib, Tshz2, Dlg2, Zfp521, Mecom, Ldlrad4, Stxbp6, Bmp6, Ier2, Elmo1, Gstt1, Fndc1, Gstm1, Ston2, Tcf7l1, Man1c1, Ccbe1, Plpp3, Rab30, Selenbp1, Fbln1, Btg2, Pcolce2,	Zbtb16, Fkbp5, B2m, Casp4, Col4a2, Ifi211, Klhl2, Birc3, Lpl, 4931406P16Rik, Ip6k2, Spon1, Marcks, Raph1, Sema6d, Vcan, Sod2, Kalrn, Fchsd2, Hipk1, Ube2q2, Aspn, Rgs7, Stim1

	B4galt1, Cdon, Fam13c, Palm, Myrip, Fam20a, Aldh1a2, Chrm3, Junb	
count	44	24
